# Halofuginone for non-hospitalized adult patients with COVID-19 a multicenter, randomized placebo-controlled phase 2 trial. The HALOS trial

**DOI:** 10.1371/journal.pone.0299197

**Published:** 2024-02-23

**Authors:** Bruno Martins Tomazini, Lucas Tramujas, Fernando Azevedo Medrado, Samara Pinheiro do Carmo Gomes, Karina Leal Negrelli, Gabriela Souza Murinize, Renato Hideo Nakagawa Santos, Bruna Martins Pereira Vianna, Bruna Fornazieri Piotto, Thabata Silva Veiga, Bianca Rodrigues do Santos, Ana Clara Peneluppi Horak, Olivia Mora Cavalcante Lemos, Marcela de Almeida Lopes, Beatriz Baptista Olicheski, Diego Lurentt Campones, Luiz Angelo Alencar Peixoto, Aline dos Anjos Chaves Basilio, Otavio Celso Eluf Gebara, Ana Tarina Alvarez Lopes, Humberto Saconato, Nanci Valeis, Tamiris Abait Miranda, Ligia Nasi Laranjeira, Eliana Vieira Santucci, Aaron Foster Carlin, Jeffrey David Esko, Phillip Leo Stephan Marie Gordts, Sotirios Tsimikas, Alexandre Biasi Cavalcanti

**Affiliations:** 1 Hcor Research Institute, São Paulo (SP), Brazil; 2 Brazilian Research in Intensive Care Network (BRICNet), São Paulo (SP), Brazil; 3 Hospital Sírio-Libanês, São Paulo (SP), Brazil; 4 Hospital do Coração (HCor), São Paulo (SP), Brazil; 5 Hospital Santa Paula, São Paulo (SP), Brazil; 6 Departments of Pathology and Medicine, University of California, San Diego, La Jolla, California, United States of America; 7 Department of Cellular and Molecular Medicine and Glycobiology Research and Training Center, University of California, San Diego, La Jolla, California, United States of America; 8 Department of Medicine, and Glycobiology Research and Training Center, University of California, San Diego, La Jolla, California, United States of America; 9 Division of Cardiovascular Medicine, Sulpizio Cardiovascular Center, University of California San Diego, La Jolla, California, United States of America; Universidad de La Sabana, COLOMBIA

## Abstract

**Background:**

Halofuginone (PJS-539) is an oral prolyl-tRNA synthetase inhibitor that has a potent *in vitro* activity against SARS-CoV-2 virus. The safety and efficacy of halofuginone in Covid-19 patients has not been studied.

**Methods:**

We conducted a phase II, randomized, double-blind, placebo-controlled, dose ranging, safety and tolerability trial of halofuginone in symptomatic (≤ 7 days), mostly vaccinated, non-hospitalized adults with mild to moderate Covid-19. Patients were randomized in a 1:1:1 ratio to receive halofuginone 0.5mg, 1mg or placebo orally once daily for 10 days. The primary outcome was the decay rate of the SARS-CoV-2 viral load logarithmic curve within 10 days after randomization.

**Results:**

From September 25, 2021, to February 3, 2022, 153 patients were randomized. The mean decay rate in SARS-CoV-2 viral load log_10_ within 10 days was -3.75 (95% CI, -4.11; -3.19) in the placebo group, -3.83 (95% CI, -4.40; -2.27) in the halofuginone 0.5mg group and -4.13 (95% CI, -4.69; -3.57) in the halofuginone 1mg group, with no statistically significant difference in between placebo vs. halofuginone 0.5mg (mean difference -0.08; 95% CI -0.82 to 0.66, p = 0.96) and between placebo vs. halofuginone 1mg (mean difference -0.38; 95% CI, -1.11; 0.36, p = 0.41). There was no difference on bleeding episodes or serious adverse events at 28 days.

**Conclusions:**

Among non-hospitalized adults with mild to moderate Covid-19 halofuginone treatment was safe and well tolerated but did not decrease SARS-CoV-2 viral load decay rate within 10 days.

## Introduction

Since the beginning of the coronavirus disease 2019 (Covid-19) pandemic several drugs with possible antiviral effects for non-hospitalized patients have been studied with different degrees of success, with some treatments decreasing the risk of severe Covid-19 [1–5]. New antiviral treatments could potentially benefit Covid-19 patients in the early phase of disease.

The severe acute respiratory syndrome coronavirus 2 (SARS-CoV-2) virus uses a complex mechanism to infect and replicate in the host cell. One important step in this process is the binding of the viral structural protein S to the domain of the angiotensin-converting enzyme 2 (ACE2) [6–8] in the host cell, using heparan sulfate as a cofactor [7]. These steps might be a target for antiviral therapies, decreasing viral entry and leading to less severe disease.

Halofuginone (PJS-539), a synthetic derivative of febrifugin [9, 10], is a molecule with anti-fibrotic, anti-angiogenic and anti-proliferative properties [9] with high lung tissue concentration after administration [11]. Halofuginone has been identified as a potent in-vitro inhibitor of SARS-CoV-2 cell adhesion dependent on protein S and heparan sulfate [12, 13]. Its mode of action involves inhibition of the prolyl-tRNA synthetase [14], a member of the aminoacyl-tRNA synthetase family of enzymes. This enzyme is essential for RNA translation, and proteins rich in proline residues are especially susceptible to inhibition, including translation of heparan sulfate proteoglycans that serve as a coreceptor for SARS-CoV-2 and RNA processing enzymes required for viral replication [14, 15].

Halofuginone was previously evaluated in phase I and phase II studies in humans for other purposes [16–18]. Based on safety, tolerability, pharmacokinetic and pharmacodynamic data of a phase I trial [16], the dosages of 0.5mg and 1mg of halofuginone were selected for further investigation. The HALOS trial (Halofuginone for SARS-CoV-2) evaluated the safety and efficacy of halofuginone in non-hospitalized adults with mild to moderate Covid-19.

## Methods

### Study design

This was a phase II, randomized, double-blind, placebo-controlled trial (Study protocol available in S1 Appendix). The study was approved by the Brazilian National Commission for Research Ethics (CONEP), and all research ethics committees at the participating sites. Written informed consent was obtained from all patients before randomization. This report follows the Consolidated Standards of Reporting Trials (CONSORT) reporting guidelines (S2 Appendix). The study was conducted in two emergency departments (Hospital do Coração—Hcor and Hospital Santa Paula) in São Paulo, Brazil. The trial was registered with ClinicalTrials.gov (NCT05008393).

### Participants

Adult patients who presented at the emergency department of the participating centres were triaged for participating in the trial (details in supplemental materials). Eligible patients were ≥18 years old, had confirmed Covid-19 by detection of SARS-CoV-2 by reverse transcription polymerase chain reaction (RT-PCR), rapid genetic test or antigen test, had mild to moderate symptoms [respiratory symptoms (cough, dyspnea, and rhinorrhea), gastrointestinal symptoms (nausea, vomiting, and diarrhea), and other symptoms (fever, muscle or joint pain, headache or fatigue)] without indication for hospitalization, had duration of symptoms attributable to Covid-19 ≤7 days, and had the ability to access the study’s online questionnaire. Key exclusion criteria were pregnancy or active lactation, estimated glomerular filtration rate less than 30 ml per minute per 1.73 m^2^, clinical history of cirrhosis, decompensated heart failure, previous participation in the study, participation in other clinical trials with antivirals for Covid-19, and high risk of bleeding (S3 Appendix). High risk of bleeding was defined as any of the following: previous intracranial hemorrhage, ischemic stroke in the past 3 months, known anatomical vascular malformation of the central nervous system, such as aneurysms or arteriovenous malformations, known malignant neoplasm of the central nervous system, metastatic solid neoplasia, significant closed head or facial trauma in the past 3 months (defined as any trauma that required medical evaluation or hospitalization), known intracranial abnormalities not listed as absolute contraindications (e.g., benign intracranial tumor), bleeding in the past 2 to 4 weeks (excluding menstrual bleeding), surgical procedure in the past 3 weeks, current use of full-dose anticoagulants (warfarin, enoxaparin or new anticoagulants) or dual antiplatelet therapy or thrombocytopenia (<100.000/mL) or INR (international standardized ratio) > 1.3.

### Interventions and randomization

Halofuginone 0.5mg/daily and 1mg/daily (PJS-539) were chosen based on the safety profile of published data [16]. In these dosages, the most common adverse effects reported were nausea and vomiting after taking the drug (S3 Appendix). Antiemetics (ondansetron or metoclopramide) were provided to all patients. In the current study, use of prophylactic antiemetics 30 to 60 minutes before taking the next study medication dose was advised if patients had at least one episode of nausea or vomit. There were no other mandatory study interventions, and all patients received standard treatment care indicated by the treating physician and institutional guidelines.

Randomizations was performed through an online web-based system(19) using computer-generated random numbers and blocks of 3 and 6, unknown to the investigators, and was stratified by center and age (< 60 and ≥ 60 years old). Eligible patients were randomized in a 1:1:1 ratio to receive halofuginone 0.5mg, halofuginone 1mg or placebo orally once daily for 10 days (S3 Appendix). In case of hospitalization, severe adverse events (death, threat to life, persistent or significant incapacity/disability, hospitalization requirement or prolongation, or any clinically significant event defined by the treating physician) or the need for anticoagulation within 10 days of randomization the study drug or placebo were stopped.

### Study assessments

Assessments included collection of demographic and baseline data at the moment of randomization, laboratory testing for the purpose of safety were performed at baseline and 10 days after randomization, and nasopharyngeal swabs for SARS-CoV-2 for viral load measurements were collected at baseline, 5 days, and 10 days after randomizations. All patients received a portable oximeter to measure daily their peripheral oxygen saturation and heart rate. An online questionnaire containing questions on clinical symptoms, clinical signs (peripheral oxygen saturation and heart rate), and use of study medication, was sent daily to all patients up to day 10. Follow-up phone calls were performed on day 14 and 28 to collect clinical data (S1 and S4 Appendices). Adverse events were assessed daily until day 10 and on the 14^th^ and 28^th^ day follow-up phone calls. All study data were collected using a secure electronic data capture system [19].

### Outcomes

The primary outcome was the decay rate of the SARS-CoV-2 viral load logarithmic curve within 10 days after randomization. Secondary outcomes were hospital admissions within 28 days, need for invasive mechanical ventilation within 28 days, time to symptoms resolution from randomization up to day 10, ordinal clinical scale of symptoms on day 14 (1. not hospitalized, without limitation of daily activities; 2. not hospitalized, with limitation of daily activities; 3. hospitalized, without the need for supplemental oxygen; 4. hospitalized, requiring supplemental oxygen; 5. hospitalized, requiring high-flow nasal oxygen therapy, non-invasive mechanical ventilation, or both; 6. hospitalized, requiring blood oxygenation through a membrane system, invasive mechanical ventilation, or both; 7. Death) [5, 20, 21], adverse events within 28 days, and bleeding events [22] within 28 days.

### Sample size

Sample size calculations were based on an average linear decay rate of SARS-CoV-2 viral load in nasopharyngeal swab of 1.7 log_10_ every 5 days [23]. Assuming this decay rate for the placebo group and considering that all selected active treatments would have a viral load decay rate of 2.2 log_10_, 150 patients with confirmed SARS-CoV-2 (50 per group) would provide the trial with 90% power to indicate that at least one of the treatments is superior to the placebo, considering a two-tailed Bonferroni adjusted significance level of 2.5%, to guarantee an overall level of significance of 5%. The sample size calculations were performed using simulations considering different standard deviations in each time step (baseline, 5 days and 10 days), and an auto-regressive correlation of 0.85. The covariance matrix was based on data on the daily SARS-CoV-2 viral load [24].

### Statistical analysis

The primary analysis followed a modified intention to treat (mITT) approach, considering only patients with positive Covid-19 laboratory diagnosis by RT-PCR and who had at least one viral load measured.

To estimate the treatment effect on the primary outcome, we used a linear mixed model with an interaction term between the group variable and time of viral load collection, with random effect for the intercept and for the viral load decay associated to each patient.

We analyzed the treatment effect on time to resolution of symptoms from randomization up to day 10, summing the number of days each patient experienced without symptoms (symptom-free days), using a proportional odds logistic regression model. We originally planned to analyze the ordinal scale on day 14 using a proportional odds logistic regression model. However, given we only had patients in categories 1 or 2 of the ordinal scale on day 14, we used a logistic regression model. Adverse events are expressed as counts and percentages and compared between groups using the Fisher test.

We performed subgroup analysis on the primary outcome testing interactions for duration of symptoms at randomization (≤ 4 days and > 4 days) and time of recruitment (until December 20^th^, 2021, and after December 20^th^, 2021). The time of recruitment analysis was chosen based on the possible onset of omicron wave in Brazil. Three sensitivity analyses on the primary outcome were performed, one using a mixed effect model with the date of nasal swab collection as a continuous second-degree polynomial variable, one using a linear mixed model considering the date of nasal swab collection as a categorical variable, both with random effect for the intercept and for the viral load decay associated to each patient, and one per protocol analysis.

For patients without viral load available for a specific timepoint, viral load data was imputed using an estimate via the chain equation method using the mice package in R [25, 26]. We chose to perform the primary outcome assessment based on multiple imputation methods to correct any viral load values due to loss of information (exam not performed), or if the exam was performed outside a 3-day window of the planned collection date. We used available data on viral load and symptoms as predictor variables. In case of missing data on symptoms up to 10 days, data was imputed using the last observation carried forward method.

A safety interim analysis was planned after 60 patients have reached the outcome of the 28-day follow-up study. However, when the 60^th^ patient reached the 28-day outcome, the total sample size had already been enrolled and the interim analysis was not performed.

All patients were analysed according to their randomization group. For the primary outcome a Bonferroni adjusted 2-sided P value of less than 0.025 was considered statistically significant. For all other outcomes no adjustments for multiplicity were performed and a 2-sided P value of less than 0.05 was considered statistically significant. The results of subgroup analyses and secondary outcomes should be interpreted as exploratory. All analyses were performed using the R software version 4.2.0 (R Core Team).

## Results

### Patients

**Fig 1:**
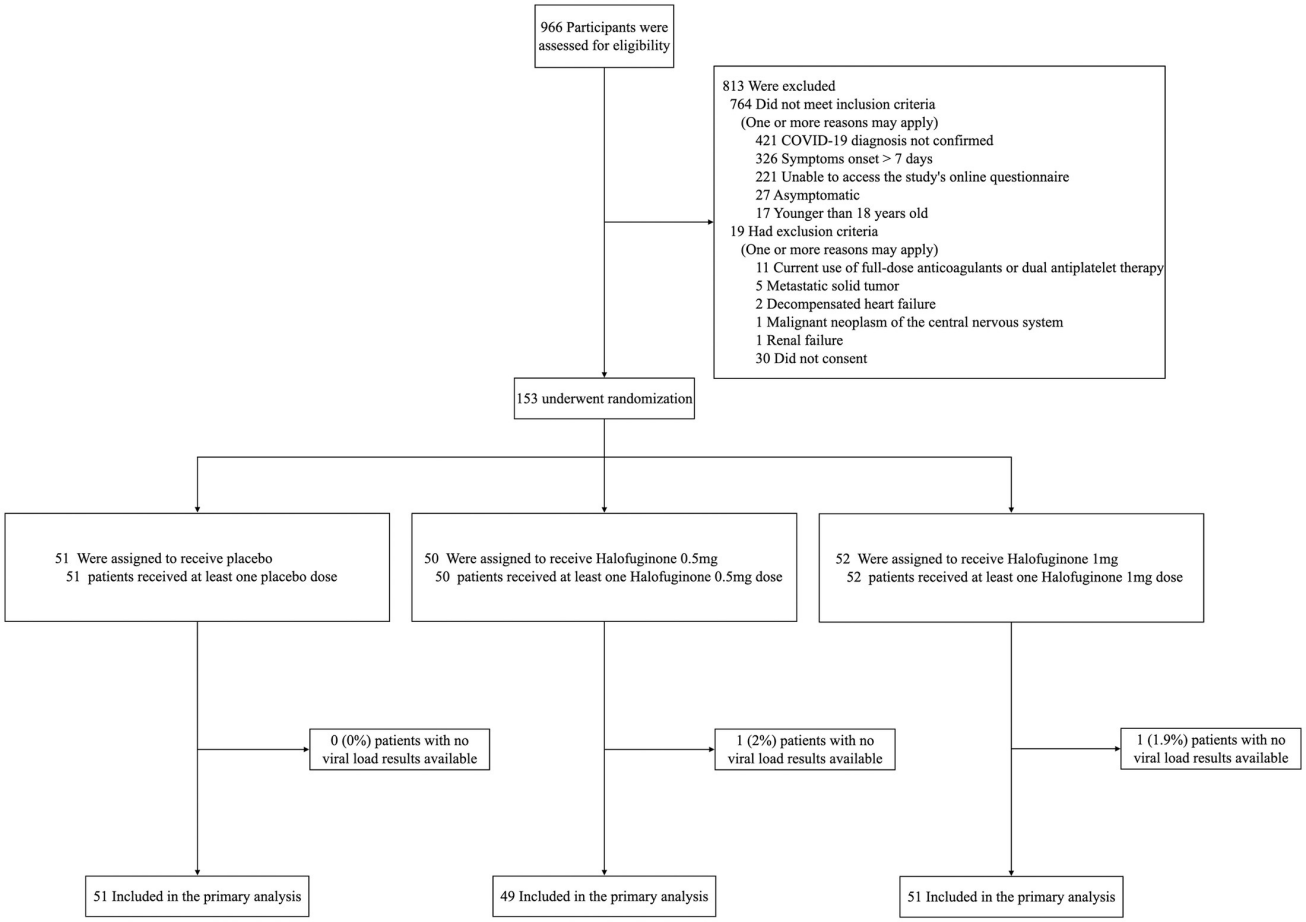
Flow of patients in Halos trial.

From September 25, 2021, to February 3, 2022, we screened 966 and randomized 153 patients. Among the patients who underwent randomization, 51 were assigned to receive placebo, 50 to receive halofuginone 0.5mg and 52 to receive halofuginone 1mg. One patient in the halofuginone 0.5mg and one patient in the halofuginone 1mg group had no viral load results available (S1 Table). A total of 151 patients were included in the mITT analysis (Fig 1). Baseline characteristics were balanced between groups, except for a lower prevalence of hypertension in the halofuginone 0.5mg group (Table 1; S2 Table).

**Table 1:** Baseline characteristics of the patients^a^.

Characteristic	Patients, No. (%)			
	Total (N = 153)	Placebo (N = 51)	Halofuginone 0.5mg (N = 50)	Halofuginone 1mg (N = 52)
**Age, y**				
**Mean ± SD**	43.3 ± 13.5	42.8 ± 13.2	43.1 ± 13	43.9 ± 14.6
**≥ 60**	22 (14.4)	7 (13.7)	8 (16)	7 (13.5)
**BMI, kg/m^2^**	27.4 ± 4.8	28.2 ± 5.3	27.2 ± 4.8	26.8 ± 4.3
**Sex at birth, n (%)**				
**Men**	64 (42.1)	20 (39)	21 (42)	23 (44.2)
**Woman**	89 (57.9)	31 (61)	29 (58)	23 (55.8)
**Underlying diseases, n (%)**				
**Hypertension**	27 (17.6)	12 (23.5)	3 (6)	12 (23.1)
**Arrhythmia**	5 (3.3)	1 (2)	1 (2)	3 (5.8)
**Obesity**	18 (11.)	7 (13.7)	6 (12)	5 (9.6)
**Diabetes**	9 (5.9)	2 (3.9)	2 (4)	5 (9.6)
**COPD**	1 (0.7)	0 (0)	0 (0)	1 (1.9)
**Current smoking**	12 (7.8)	5 (9.8)	3 (6)	4 (7.7)
**Previous smoking**	15 (9.8)	5 (9.8)	5 (10)	5 (9.6)
**COVID vaccinated, n (%)**	150 (98)	49 (96.1)	50(100)	51 (98.1)
**Vaccine Booster dose, n (%)**	74 (48)	26 (53)	28 (56)	20 (40)
**Laboratory variables, median (IQR)**				
**Hemoglobin g/dL**	14.3 (13.5–15.2)	14.4 (13.6–15.6)	13.9 (13.4–14.9)	14.2 (13.5–14.9)
**White blood cell count ×10^9^/L**	6.2 (5.0–7.9)	6.3 (5.2–8.4)	5.9 (4.9–7.3)	6.3 (5.0–7.6)
**Lymphocyte count ×10^9^/L**	1.9 (1.5–2.3)	2.1 (1.8–2.5)	1.8 (1.4–2.2)	1.8 (1.6–2.2)
**Platelets count ×10^9^/L**	237 (189–273)	233 (191–273)	237 (190–260)	240 (185–276)
**Creatinine mg/dL**	0.85 (0.74–0.97)	0.83 (0.72–0.96)	0.84 (0.75–0.96)	0.87 (0.75–0.98)
**C-reactive protein mg/L**	10.1 (6.5–21.1)	9.85 (6.43–20.3)	11.7 (8.25–26.15)	8 (5.9–18.3)
**Days from symptom’s onset to randomization, n (%)**				
**≤ 4 days**	64 (41.8)	21 (41.1)	22 (44)	21 (40.3)
**Symptoms, n (%)**				
**Cough**	131 (85.6)	45 (88.2)	42 (84)	44 (84.6)
**Dyspnea**	36 (23.5)	14 (27.5)	10 (20)	12 (23.1)
**Rhinorrhea**	121 (79.1)	39 (76.5)	40 (80)	42 (80.8)
**Nausea**	23 (15)	9 (17.6)	5 (10)	9 (17.3)
**Vomiting**	1 (0.7)	0 (0)	1 (2)	0 (0)
**Diarrhea**	28 (18.3)	12 (23.5)	10 (20)	6 (11.5)
**Fever**	68 (44.4)	25 (49)	21 (42)	22 (42.3)
**Muscle or joint pain**	103 (67.3)	42 (82.4)	28 (56)	33 (63.5)
**Headache**	111 (72.5)	41 (80.4)	35 (70)	35 (67.3)
**Fatigue**	93 (60.8)	38 (74.5)	26 (52)	29 (55.8)
**Clinical variables**				
**Temperature, ºC**	36.6 ± 0.5	36.6 ± 0.5	36.5 ± 0.6	36.6 ± 0.5
**Systolic blood pressure, mmHg**	122.7 ± 15.3	124 ± 18.3	121.3 ± 15.2	123 ± 13.1
**Diastolic blood pressure, mmHg**	80.2 ± 11	80.9 ± 14.8	80.2 ± 10.2	79.8 ± 8.3
**Heart rate, bpm**	80.2 ± 14.5	80.5 ± 13.5	82.7 ± 15.1	77.5 ± 14.5
**Respiratory rate, ipm**	16.8 ± 1.4	16.8 ± 1.3	16.7 ± 1.6	16.8 ± 1.4
**Peripheral oxygen saturation, %**	97.9 ± 1.4	97.4 ± 1.7	97.9 ± 1.4	98.1 ± 1.3
**Viral load, log_10_ copies per milliliter, median (IQR)**	5 (4.1–5.8)	5 (3.9–5.8)	5.3 (4.3–5.9)	4.9 (4.1–5.8)
**Missing viral load, n (%)**	5 (3.2)	1 (2)	3 (6)	1 (1.9)

Abbreviations: IQR, interquartile range; BMI, body mass index; bpm, beats per minute; ipm, incursions per minute.

^a^ Continuous variables are presented as mean ± SD unless otherwise indicated

### Study drug adherence

All patients took at least one dose of halofuginone or placebo. The number of patients who confirmed have taken all 10 doses in placebo group were 33 (64.7%), in the halofuginone 0.5mg group were 31 (62%), and halofuginone 1 mg group were 29 (55.8%). In total, patients who confirmed have taken 8 doses in the placebo group were 46 (90.2%), in the halofuginone 0.5mg group were 42 (84.0%), and in the halofuginone 1mg group were 39 (75.0%). The main cause for non-adherence was treatment suspension due to adverse events, with a total of 4 patients (8.0%) in the halofuginone 0.5mg group, 9 patients (17.3%) in the halofuginone 1mg group and none in the placebo group (p = 0.003). Data on adherence was missing in 34 of the 510 total daily doses in the placebo group (6.6%), 36 of the 500 total daily doses in the halofuginone 0.5mg group (7.2%), and 31 of the 520 total daily doses in the halofuginone 1mg group (5.9%). More information on drug adherence available in supplement (S3A–S3C Table).

### Primary analyses

The mean decay rate in SARS-CoV-2 viral load log_10_ at 10 days was -3.75 (95% CI, -4.11; -3.19) in the placebo group, -3.83 (95% CI, -4.40; -2.27) in the halofuginone 0.5mg group and -4.13 (95% CI, -4.69; -3.57) in the halofuginone 1mg group, with no statistically significant difference between placebo vs. halofuginone 0.5 mg (p = 0.96) and between placebo vs. halofuginone 1mg (p = 0.41) (Table 2).

**Table 2:** Study outcomes.

Outcomes	Placebo (95% CI)	Halofuginone 0.5mg (95% CI)	Halofuginone 1mg (95% CI)	Effect Statistic	Halofuginone 0.5mg vs Placebo		Halofuginone 1mg vs Placebo	
					Estimate (95% CI)	p-value	Estimate (95% CI)	p-value
**Primary outcome**								
**SARS-CoV-2 viral load log_10_ mean decay rate at 10 days**	-3.75 (-4.31; -3.19)	-3.83 (-4.4; -3.27)	-4.13 (-4.69; -3.57)	MD	-0.08 (-0.82; 0.66)	0.96	-0.38 (-1.11; 0.36)	0.41
**Secondary outcomes**								
**Hospital admissions within 28 days** ^a^	0/51 (0%)	0/50 (0%)	2/52 (3.8%)	-	-	-	-	-
**Need for invasive mechanical ventilation within 28 days**	0/51 (0.0%)	0/50 (0.0%	0/52 (0.0%)	-	-	-	-	-
**Symptoms–free days up to day 10, median [IQR]**	2 [0–5.5]	2 [0–5]	1.5 [0–3.2]	POR^§^	0.90 (0.44; 1.84)	0.77	1.01 (0.51; 2.02)	0.97
**Ordinal clinical scale of symptoms on day 14** ^b^								
** Not hospitalized without limitations of daily activities (Ref.)**	46/51 (90.2%)	47/50 (94%)	50/52 (96.2%)	-	-	-	-	-
** Not hospitalized but with limitations of daily activities**	5/51 (9.8%)	3/50 (6%)	2/52 (3.8%)	OR	0.59 (0.12; 2.53)	0.48	0.37 (0.05; 1.80)	0.25
**Exploratory outcomes**								
**Respiratory Symptoms** ^c^ –**free Days Up to Day 10**	3 [0–7]	5 [1–7]	5.5 [3–9]	POR^§^	1.47 (0.73; 2.97)	0.28	2.16 (1.09; 4.31)	0.03
**Gastrointestinal Symptoms ^d^–free Days Up to Day 10**	9 [7.5–10]	8 [6.2–9]	7.5 [4–9]	POR^§^	0.39 (0.19; 0.8)	0.01	0.19 (0.09; 0.39)	<0.001
**Other Symptoms ^e^–free Days Up to Day 10**	6 [2–8.5]	5 [2–8]	4 [1.8–8]	POR^§^	0.80 (0.40; 1.57)	0.51	0.61 (0.31; 1.2)	0.15

Abbreviations: CI, confidence interval; MD, mean difference; POR, proportional odds ratio; OR, odds ratio, IQR, interquartile range, Ref, reference.

^a^ Two patients were admitted to the hospital during the study, both from the halofuginone 1mg group. One patient was admitted due to bacterial pneumonia secondary to Covid-19, and one patient due to acute cholecystitis. Both patients were discharged alive without further complications.

^b^ As of day 14, none of the patients were hospitalized. All patients were in only two categories of the ordinal scale (1. not hospitalized, without limitation of daily activities; 2. not hospitalized, with limitation of daily activities)

^c^ Respiratory symptoms included: cough, dyspnea, and rhinorrhea

### Secondary analyses

We found no difference between groups in hospital admissions within 28 days and none of the study patients underwent invasive mechanical ventilation during the study period (Table 2).

**Fig 2:**
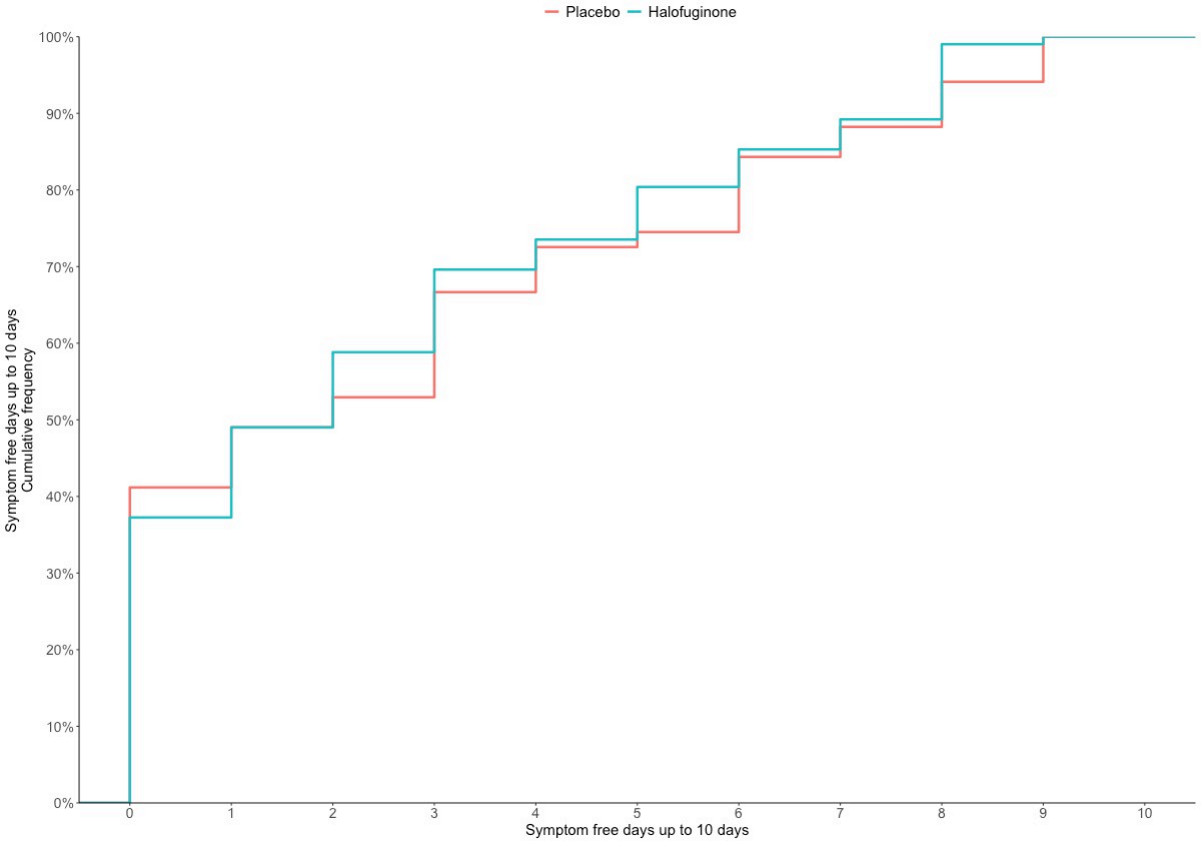
Symptoms free-days up to day 10.

There was no statistically significant difference in symptom-free days up to day 10 [median 2 days; IQR 0–5.5 days for the placebo group; median 2 days; IQR 0–5 days for the halofuginone 0.5mg group; median 1.5 days; IQR 0–3.2 days for the halofuginone 1mg group; (Table 2 and Fig 2).

There was no difference in the clinical ordinal scale of symptoms on day 14, with patients of all groups in only two categories of the ordinal scale (1. not hospitalized, without limitation of daily activities; 2. not hospitalized, with limitation of daily activities (Table 2).

### Adverse events

Participants in the halofuginone groups had more adverse events reported than those in the placebo group (64.7% in placebo vs. 92% in halofuginone 0.5mg vs. 92.3% in halofuginone 1mg, p<0.001). Specifically, patients in halofuginone groups experienced more nausea (19.6% in placebo vs. 68% in halofuginone 0.5mg vs. 76.9% in halofuginone 1mg, p<0.001), vomiting (7.8% in placebo vs. 48% in halofuginone 0.5mg vs. 67.3% in halofuginone 1mg, p<0.001) and constipation (2% in placebo vs. 8% in halofuginone 0.5mg vs. 17.3% in halofuginone 1mg, p = 0.02). There was no difference on bleeding episodes or serious adverse events at 28 days (Table 3 and S4 Table). Two patients in the halofuginone 1mg group had serious adverse events, both due to need for hospitalization (one was admitted due to bacterial pneumonia secondary to Covid-19 on study-day 4, and one patient due to acute cholecystitis on study-day 14).

**Table 3:** Adverse events ^a^.

	Placebo (N = 51)	Halofuginone 0.5mg (N = 50)	Halofuginone 1mg (N = 52)	p-value
**Any Adverse Events, n (%)**	33 (64.7)	46 (92)	48 (92.3)	<0.001
**Nausea, n (%)**	10 (19.6)	34 (68)	40 (76.9)	<0.001
**Vomiting, n (%)**	4 (7.8)	24 (48)	35 (67.3)	<0.001
**Constipation, n (%)**	1 (2)	4 (8)	9 (17.3)	0.02
**Bleeding** ^b^, **n (%)**	1 (2)	2 (4)	1 (1.9)	0.69
**Serious adverse events** ^c^, **n (%)**	0/51 (0)	0/50 (0)	2/52 (3.8)	0.33

^a^ All-adverse events were evaluated up to day 28.

^b^ All bleeding episodes were classified as minor.[23] Bleeding episode in the placebo group: 1 episode of nasal bleeding on study day 14; Bleeding episodes in the halofuginone 0.5mg group: 1 episode of gingival bleeding on study day 1, and 1 episode of increased menstrual bleeding on study day 9; Bleeding episode in the halofuginone 1mg group: 1 episode of intermenstrual bleeding on study day 14.

^c^ Serious adverse events occurred in two patients and were due to hospitalizations. One patient was admitted due to bacterial pneumonia secondary to Covid-19 on study-day 4, and one patient due to acute cholecystitis on study-day 14. Both patients were discharged alive without further complications.

There was no statistically significant difference between groups in the laboratory variables collected at day 10, except for white blood cell count halofuginone 1mg group (S5A and S5B Table).

### Subgroup and exploratory analyses

Patients in the halofuginone 1mg group had more respiratory symptoms-free days up to day 10 than placebo (median 5.5 days; IQR 3–9 days vs. 3 days; IQR 0–7 days; p = 0.03). There was no statistically significant difference in respiratory symptoms-free days between placebo and halofuginone 0.5mg. (Table 2, S6 Table and S1–S4 Figs). Patients in the placebo group had more gastrointestinal symptoms-free days up to day 10 than both halofuginone 0.5mg and halofuginone 1mg [median 9 days; IQR 7.5–10 days for the placebo group; median 8 days; IQR 6.2–9 days for the halofuginone 0.5mg group; median 7.5 days; IQR 4–9 days for the halofuginone 1mg group; (p = 0.01 placebo vs. halofuginone 0.5mg; p<0.001 placebo vs. halofuginone 1mg)] (S6 Table and S5–S9 Figs).

In subgroup analysis, tests for interaction were not statistically significant for the subgroups defined by symptoms duration at randomization and time of recruitment (S10 Fig).

**Fig 3:**
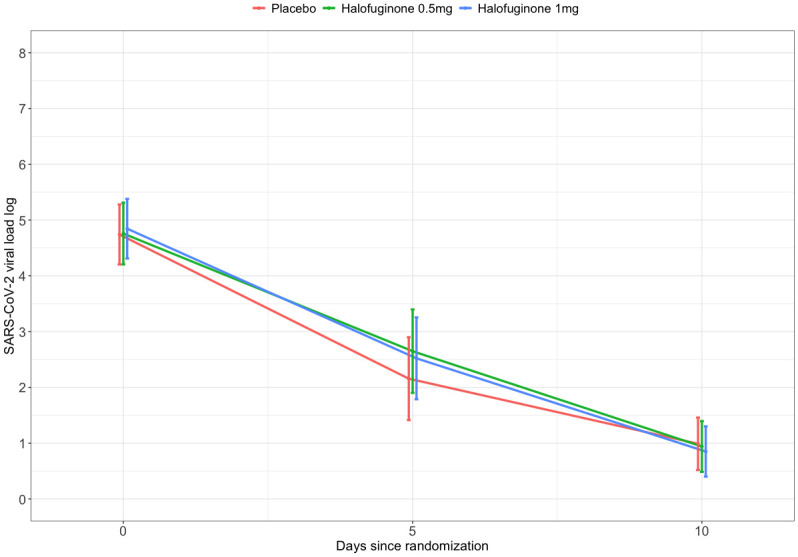
Mean SARS-CoV-2 viral load log 10 between groups.

The sensitivity analyses on the primary outcome with the date of nasal swab collection as a continuous variable, as a categorical variable and the per protocol analysis showed no difference in the outcome (S7 Table). Fig 3 shows the mean SARS-CoV-2 viral load log_10_ between groups considering swab collection as a categorical variable.

Post-hoc analyses showed no significant difference in symptoms at 14 and 28 days (S8 Table).

## Discussion

This phase-2 randomized clinical trial evaluated the safety and efficacy of two regimens of halofuginone on viral load and other clinical outcomes in non-hospitalized adults with mild to moderate Covid-19. We found no statistically significant difference on the primary endpoint of viral load decay rate within 10 days between groups. Among the secondary outcomes, patients in the halofuginone groups experienced more adverse events, mostly due to nausea and vomiting when compared to placebo. We found no difference in bleeding and other adverse events.

This was the first clinical trial evaluating safety and efficacy of halofuginone in Covid-19 patients. In vitro data showed that halofuginone inhibits heparan sulfate biosynthesis at both translational and transcriptional level, [12] therefore reducing spike protein binding of SARS-CoV-2 a critical step in host cell infection. Also, by inhibiting the host prolyl-tRNA synthetase, halofuginone decreases SARS-CoV-2 replication [12]. We found no evidence of effect of halofuginone on the SARS-CoV-2 viral load within 10 days. However, the method used for viral load quantification, by E-gene targeting [27], might not differentiate between dead and live viruses [28]. Also, previous studies showed that even for clinically effective COVID-19 treatments, the changes in viral load might not be significant [4]. Given the safety profile of halofuginone showed in our study, a possible effect on decreasing respiratory symptoms, associated with the fact that even in effective Covid-19 treatments the viral load might not correlates with clinical outcomes, a phase 3 clinical trial to evaluate the effect of halofuginone in clinical outcomes might be warranted.

We found that patients in the halofuginone 1mg group had more respiratory symptoms free-days than placebo at day 10, mostly driven by cough reduction. Given heparan sulfate proteoglycan production might be associated with respiratory secretions and cough [29], the decrease in cough might be explained by the inhibition of heparan sulfate proteoglycan biosynthesis by halofuginone. However, respiratory symptoms-free days was a post-hoc exploratory outcome, and one cannot exclude random error as explanation for this finding.

A previous phase I trial showed that the most common adverse effects of halofuginone were nausea, vomiting, and possibly increased risk of bleeding [16]. Our study showed that patients receiving halofuginone experienced more nausea and vomiting during the intervention period, with a possible dose response relationship. Both symptoms ceased after the intervention period. In our study, we used a racemic mixture of halofuginone, however, data suggests the dextro-halofuginone enantiomer has higher biological effect than the levo-halofuginone enantiomer [30], therefore, using exclusively a dextro-enantiomer would allow for lower dosages and possibly lower side effects.

One important finding was that halofuginone did not increased risk of bleeding nor lead to alterations in the coagulation profile compared to placebo during the study period. Differently from the de Jonge study [16], which included cancer patients with metastasis and was not placebo controlled, we excluded patients with high risk of bleeding.

This study has limitations. First, it was designed as a phase II study with small sample size and without statistical power for assessing efficacy on clinical outcomes. Second, viral load quantification using E-gene targeting might not be an optimal outcome measure given the limitations already discussed. Third, adherence to ≥80% of study treatment was 84.0% and 75.0% in the halofuginone 0.5mg group and halofuginone 1mg groups, respectively, which reflects higher treatment suspension rates possibly due to study drug intolerance and might have impacted the results.

## Conclusions

In conclusion, we found that among non-hospitalized adults with mild to moderate Covid-19 halofuginone treatment is safe but did not decrease SARS-CoV-2 viral load decay rate within 10 days. However, improvement in respiratory symptoms-free days was observed and is likely dose-dependent. Further clinical trials are warranted to evaluate the potential use of halofuginone in Covid-19.

## Supporting information

S1 Appendix Study protocol. (PDF)

S2 Appendix CONSORT checklist. (DOC)

S3 Appendix Additional information on methods. (DOCX)

S4 Appendix Model of follow-up telephone interviews. (PDF)

S1 Table Viral load measurements with available results in each group. (DOCX)

S2 Table Additional data on baseline characteristics. (DOCX)

S3 Table A. Treatment adherence in the placebo group. B. Treatment adherence in the halofuginone 0.5mg group. C. Treatment adherence in the halofuginone 1mg group.
(ZIP)

S4 Table Adverse Events at day 28. (DOCX)

S5 Table A. Laboratory variables on day 10. B. Laboratory variables within groups between baseline and day 10.
(ZIP)

S6 Table Symptoms-free days up to day 10. (DOCX)

S7 Table Sensitivity analysis for the primary outcome. (DOCX)

S8 Table Post-hoc analysis. (DOCX)

S1 Fig Respiratory symptoms-free days up to day 10. (TIF)

S2 Fig Cough-free days up to day 10. (TIF)

S3 Fig Dyspnea-free days up to day 10. (TIF)

S4 Fig Rhinorrhea-free days up to day 10. (TIF)

S5 Fig Gastrointestinal symptoms-free days up to day 10. (TIF)

S6 Fig Nausea-free days up to day 10. (TIF)

S7 Fig Vomit-free days up to day 10. (TIF)

S8 Fig Diarrhea-free days up to day 10. (TIF)

S9 Fig Other symptoms-free days up to day 10. (TIF)

S10 Fig Subgroup analysis. (TIF)
