# Supplementary material for: Halofuginone for non-hospitalized adult patients with COVID-19 a multicenter, randomized placebo-controlled phase 2 trial. The HALOS trial
Source: PLoS One. 2024 Feb 23;19(2):e0299197. doi: 10.1371/journal.pone.0299197 (PMC10889621; doi:10.1371/journal.pone.0299197)
Supplement: S1 Appendix — (PDF) [file pone.0299197.s001.pdf]

Halofuginone for adult patients with SARS-Cov-2: Multicentre, phase 2, randomized, double-blind, placebo-controlled clinical trial.

HALOfuginone for SARS-CoV-2: HALOS trial.

**National coordinator:**	Research Institute of Hospital do Coração (IP-HCor)<br>Abílio Soares Street, 250 - Paraíso<br>ZIP CODE: 04005-000 São Paulo, SP – Brazil<br>Phone: 11 3886 4693<br>Fax: 11 3886 4695
**Coordinating center investigators:**	**Principal Investigator:**<br>Dr. Bruno Martins Tomazini<br>**Sub-investigators:**<br>Dr. Fernando G. Zampieri<br>Dr. Israel Maia<br>Dr. Alexandre Biasi Cavalcanti
**Sponsor:**	Associação Beneficente Síria - HCor
**Financial support:**	CpviCept Therapeutics, Inc.
**Version:**	3.0 of September 07, 2021

Conformity Declaration

This document is a protocol for a research project. This study will be conducted in accordance with all the stipulations of this protocol, the current regulations of the CEP/CONEP System and the guidelines established by the Document of the Americas and the Guide for Good Clinical Practice (E6/R2) of the ICH - International Council for Harmonization of Technical Requirements for Pharmaceuticals for Human Use.

SYNOPSIS

**Title**	Halofuginone for adult patients with SARS-Cov-2: Multicentre, phase 2, randomized, double-blind, placebo-controlled clinical trial.
**Public title**	HALOfuginone for SARS-CoV-2: HALOS trial
**Registration and identification number of the study:**	NCT05008393
**Primary registration date:**	17/08/2021
**Coordinating Center**	Research Institute of Hospital do Coração (IP-HCor)<br>Abílio Soares Street, 250 – Paraíso<br>ZIP CODE: 04005-000 São Paulo, SP – Brazil<br>Phone: 11 3886 4693
**Contact for scientific consultations**	Dr. Bruno Martins Tomazini<br>btomazini@hcor.com.br
**Principal Sponsor**	Associação Beneficente Síria - HCor
**Financial support**	CoviCept Therapeutics
**Health condition(s) or problem(s) studied:**	SARS-CoV-2 infection
**Study design**	Multicentre, phase 2, randomized, double-blind, placebo-controlled clinical trial.
**Objective**	To evaluate the effectiveness of the use of halofuginone in the decay rate of the SARS-CoV-2 viral load logarithmic curve obtained via nasopharyngeal swab between baseline, fifth and tenth day after randomization.
**Eligibility**	**Inclusion Criteria:**<br>1. Adult patients (age ≥ 18 years);<br>2. COVID-19 diagnosis confirmed by:<br>a. Detection of SARS-CoV-2 by RT-PCR, or<br>b. Rapid genetic or antigen tests registered by ANVISA;<br>3. Mild or moderate symptoms without indication for hospitalization;<br>4. Symptoms started seven days ago or less;

	5. Be able to access the study's online questionnaire.<br>**Exclusion Criteria:**<br>1. Pregnant or lactating women;<br>2. Known allergy or hypersensitivity to the study drug;<br>3. Patients at high risk of bleeding, defined by:<br>a. Previous Intracranial hemorrhage,<br>b. Ischemic stroke in the last 3 months,<br>c. Anatomical vascular alteration of the central nervous system known, such as aneurysms or arteriovenous malformations,<br>d. Malignant neoplasm of the central nervous system known,<br>e. Metastatic solid neoplasia,<br>f. Significant closed head or facial trauma in the last 3 months,<br>g. Intracranial abnormalities not listed as absolute contraindications (e.g., benign intracranial tumor),<br>h. Bleeding in the last 2 to 4 weeks (excluding menstrual bleeding),<br>i. Major surgery in the last 3 weeks,<br>j. Current use of full-dose anticoagulants or dual antiplatelet therapy,<br>k. Thrombocytopenia (<100,000/mL) or INR (international standardized ratio) > 1.3;<br>4. Renal failure, defined as glomerular filtration rate (GFR) estimated by the formulas MDRD or CKD-EPI <30mL/min/1.73m^2;<br>5. Previous participation in the study;<br>6. History of liver disease (cirrhosis) reported by the patient or in medical records, presence of esophageal varices or presence of ascites;

	7. Decompensated heart failure, defined by the presence of dyspnea attributed to cardiac cause, edema of the lower limbs, crackles on pulmonary auscultation or pathological jugular turgency.<br>8. Participation in other clinical trials with antivirals in COVID-19
**Randomization:**	Patients will be randomized in a 1:1:1 ratio via an online electronic form available 24 hours a day.
**Intervention:**	**Control:**<br>• Oral placebo once a daily, for 10 days;<br>**Interventions:**<br>• Halofuginone 1mg orally once daily, for 10 days;<br>• Halofuginone 0.5mg orally once daily, for 10 days.
**Data collect**	Clinical data will be collected from the day of the patient's inclusion in the study on day 1 (baseline) and daily via an online questionnaire filled by the patient until the tenth day. Follow-up calls will be made by the participating center team on the days 14 and 28 to obtain vital status and other clinical data.
**Collection of biological material**	Blood collections of the following blood laboratory tests will be performed on the day of inclusion in the study (baseline), and on the tenth day: Complete blood count (CBC), urea, creatinine, alkaline phosphatase, gamma-GT, aspartate aminotransferase, alanine aminotransferase, direct and indirect bilirubin, C-reactive protein, prothrombin time with INR, activated partial thromboplastin time, fibrinogen, sodium and potassium. On the fifth and tenth day, 1 tube will be collected to measure the serum level of halofuginone.

	Nasopharyngeal swabs for SARS-CoV-2 will be collected on days 1 (baseline), 5 and 10.
**Sample size**	A total of 150 patients with confirmed SARS-CoV-2 (50 per arm) have 90% power to detect a drop in viral load via 2.2 log^{10} nasopharyngeal swab from day 1 (baseline) for days 5 and 10 between the intervention and placebo groups
**Participating centers**	2 to 5 hospitals in Brazil
**Primary Outcome**	Slope of the logarithmic curve of the SARS-CoV-2 viral load obtained via nasopharyngeal swab between the baseline, fifth and tenth days after randomization.
**Secondary Outcomes**	1. Hospital admission between randomization and the day 28;<br>2. Need for invasive mechanical ventilation between randomization and day 28;<br>3. Time to resolve symptoms until the tenth day;<br>4. Ordinal clinical scale of symptoms on 14th (1. not hospitalized, without limitation of daily activities; 2. not hospitalized, with limitation of daily activities; 3. hospitalized, without the need for supplemental oxygen; 4. hospitalized, requiring supplemental oxygen; 5 hospitalized, requiring high-flow nasal oxygen therapy, non-invasive mechanical ventilation, or both; 6. hospitalized, requiring blood oxygenation through a membrane system, invasive mechanical ventilation, or both; 7. Death);
**Safety outcomes**	1. Grade 1, 2, 3 or 4 adverse events, which were not presented when the patient entered the study. Defined by the AIDS division (DAIDS) *Table for grading the severity of adult and*

	pediatric adverse events (corrected version 2.1, July 2017);<br>2. Bleeding<br>a. Major bleeding,<br>b. Clinically relevant non-major bleeding,<br>c. Minor bleeding;<br>3. Serious Adverse Event;<br>4. Discontinuation of study drug-related treatment.
**Statistical analysis**	The analyses will follow the intention to treat principle. The primary outcome will be a comparison of the viral load logarithm decay rates via RT-PCR to SARS-CoV-2 in 10 days between treatment groups compared to control. A linear mixed model will be used, assuming random intercept and slope for patients. The viral load measurements will be performed 5 and 10 days after randomization.<br>If the RT-PCR exam is not performed for any reason, imputation will be performed using only an estimate via the chain equation method using the *mice* package. We chose to perform the primary outcome assessment based on multiple imputation methods to correct any viral load values due to loss of information due to mortality, however, based on data by Cavalcanti et al. (2020), the expected mortality during the period of exam collection is minimal (<3%).
**Data and security monitoring committee**	An external and independent data monitoring committee (DMC) will assess safety outcomes and primary outcomes when 60 patients reach the primary assessment of the study outcome. The DMC will

	consider safety events to provide a recommendation to continue inclusion or discontinue the study.

PROTOCOL INFORMATION

**Version:**	1.0
**Date:**	May 18, 2021
**Protocol amendment number:**	Initial submission
**Authors:**	Bruno M. Tomazini<br>Fernando G. Zampieri

	Israel Maia<br>Alexandre Biasi Cavalcanti
**Version:**	2.0
**Date:**	July 26, 2021
**Protocol amendment number:**	1
**Authors:**	Bruno M. Tomazini<br>Fernando G. Zampieri<br>Israel Maia<br>Alexandre Biasi Cavalcanti
**Version:**	3.0
**Date:**	September 07, 2021
**Protocol amendment number:**	2
**Authors:**	Bruno M. Tomazini<br>Fernando G. Zampieri<br>Israel Maia<br>Alexandre Biasi Cavalcanti

TABLE OF CONTENTS

1. INTRODUCTION 13

2. OBJECTIVES.. 14

2.1 PRIMARY OBJECTIVE.. 14

2.2 Secondary objective 15

3. METHODS: RESEARCH SUBJECTS, INTERVENTIONS AND OUTCOMES 15

3.1 Study design 15

3.2 Study locations 15

3.3 Recruitment plan 15

3.4 Eligibility Criteria 16

3.4.1 Inclusion Criteria 16

3.4.2 Exclusion Criteria 16

3.5 Procedures for COVID-19 diagnosis 17

3.6 Procedures for verifying the eligibility criteria 18

3.7 Contraception in the study patients 18

3.8 Study Visits 18

3.8.1 Day 1 - baseline 18

3.8.2 Day 5 – At home 19

3.8.3 Day 10 – At home 19

3.8.4 Day 14 and day 28 - follow-up via phone call 19

3.9 Allocation concealment and randomization 20

3.10 Interventions 21

3.10.1 Treatment 21

3.10.2 Additional study interventions 21

3.10.3 Treatment interruption 22

3.10.4 Halofuginone and dosage justification 22

3.10.5 Coding of study medication 25

3.10.6 Emergency break of the designated treatment code 26

3.10.7 Adhesion 27

3.10.8 COVID-19 negative confirmatory exam 28

3.10.9 Study Helpline 28

3.10.10 Losses to follow-up 28

3.11 Collection, use and storage of biological material 29

3.11.1 Blood Tests 29

3.11.2 hCG Urine Test 29

3.11.3 Nasopharyngeal swab ... *29*
3.11.4 Locals for processing, storage and performance of laboratory tests ... *30*
3.11.5 Clinical data collection ... *31*
4. OUTCOMES ... **32**
4.1 PRIMARY OUTCOME ... 32
4.2 SECONDARY OUTCOMES ... 33
4.3 SECONDARY SAFETY OUTCOMES ... 33
5. SAMPLE SIZE ... **34**
6. STATISTICAL ANALYSIS ... **34**
6.1 OUTCOMES ... 34
6.1 SAFETY INTERIM ANALYSIS ... 35
7. DATA QUALITY CONTROL ... **35**
7.1 DATA QUALITY ASSURANCE ... 35
8. ETHICAL ASPECTS AND GOOD CLINICAL PRACTICE ... **36**
8.1 STUDY APPROVAL ... 36
8.2 INFORMED CONSENT PROCESS ... 37
8.3 CRITERIA FOR DISCONTINUING STUDY PARTICIPATION ... 38
8.4 RISKS AND DISCOMFORTS ... 39
8.5 BENEFITS FOR PARTICIPANTS ... 40
8.6 REPORTING ADVERSE EVENTS ... 41
8.6.1 Adverse Events (AE) ... *41*
8.6.2 Serious Adverse Events (SAE) ... *42*
8.6.3 Direct or indirect pregnancy ... *43*
8.7 CONFIDENTIALITY ... 43
9. AUXILIARY STUDIES ... **44**
10. DATABASE LOCK AND CLINICAL STUDY SUPERVISION ... **44**
11. STUDY ORGANIZATION ... **44**
11.1 STUDY COORDINATION ... 44

11.2 Clinical study monitoring 45
11.3 Interruption of clinical study 46

12. DISSEMINATION OF RESULTS 46

13. ADMINISTRATIVE ASPECTS 47

13.1 The role of COVICEPT Therapeutics 47
13.2 Administrative and legal obligations 47
13.3 Status of sanitary regularization of the Product Under Investigation 47
13.4 Diverse 48

14. REFERENCES 49

1. INTRODUCTION

COVID-19, a disease caused by coronavirus 2 (SARS-CoV-2) became a pandemic in March 2020.[1] Clinically, COVID-19 ranges from asymptomatic disease in some to severe forms. However, until now, only the use of corticosteroids in selected patients[2] has been shown to improve clinical outcomes in patients with COVID-19.

SARS-CoV-2 is a beta-coronavirus enveloped with its genetic material (RNA) encoding 9860 amino acids.[3] The SARS-CoV-2 gene fragments encode both structural and non-structural proteins.[3] Among structural proteins, protein S (spike), protein E (envelope), and protein M (membrane) are part of the viral envelope.[4] It is known that the mechanism of binding and entry of the virus into the cell is mediated by protein S.[5] Basically, the initial stage of the infection process begins with the binding of the virus to the host cell via the target receptor. The S1 subunit of the SARS-CoV-2 protein S has a domain that binds to the domain of the angiotensin-converting enzyme 2 (ECA2).[5-7]

It is known that heparan sulfate (HS), a negatively charged polysaccharide that is bound to proteoglycans in the cell membrane or extracellular matrix, is a cofactor for SARS-CoV-2 infection.[6] The protein S, specifically its S1 subunit, from SARS-CoV-2 interacts with HS which functions as a co-receptor facilitating the binding between protein S and ECA2.[5 6] Blocking these interactions between protein S, ECA2 and HS attenuates the binding and infection of SARS-CoV-2[6] and are potential targets for antivirals and vaccines against COVID-19. [3 8 9]

Halofuginone, a synthetic derivative of febrifugin (an alkaloid derived from *Dichroa febrifuga*, one of the 50 fundamental herbs of traditional Chinese medicine)[10 11] is a molecule with anti-fibrotic, anti-angiogenesis and anti-proliferative properties in preclinical studies[10] and has been tested in some phase I and phase II studies. [12-14] Through screening of epigenetic and translational regulatory compounds, halofuginone has been identified as a potent inhibitor of SARS-CoV-2 cell adhesion dependent on protein S and heparan sulfate (HS) (Sandoval, et.al - unpublished data - available in Annex I). In cultures of human bronchial epithelium infected with SARS-CoV-2, halofuginone in concentrations of 10nM and 100nM significantly reduced the number

of cells infected by SARS-CoV-2 without affecting cell viability (Sandoval, et.al - unpublished data - available in Annex I).

Halofuginone is known to be a potent inhibitor of “prolyl-tRNA synthetase” (PRS).[11] Inhibition of PRS suppresses the translation of proline-rich proteins, while having a minimal effect on general protein synthesis.[11] Sandoval and collaborators (unpublished data - available in Annex I) demonstrated that halofuginone inhibits SARS-CoV-2 viral replication, showing a reduction of about 1000 times in the number of virions secreted in cells treated with halofuginone, as well as a drop in mRNA intracellular protein S of SARS-CoV-2 in more than 20,000 times, with an average inhibitory concentration (IC50) of 34.9nM.

Commercial halofuginone is a racemic mixture of dextrogyrous and levogyrous enantiomers.[15] It was observed that the enantiomer active in the viral inhibition of SARS-CoV-2 is the dextrogyrus, with an IC50 of 12nM. Oral halofuginone has good bioavailability, with a maximum serum concentration (Cmax) of 0.54ng/mL (~1.3nM) after oral 0.5mg and Cmax 3.09ng/mL (~7.4nM) after oral 3.5mg in a study phase I.[12] Although with these doses tested it is not possible to obtain plasma levels significantly above the IC50 demonstrated by Sandoval et al, studies show that the concentration of halofuginone in lung tissue can be up to 87 times higher than in plasma.[16]

Thus, we hypothesized that the antiviral effect of halofuginone is both by inhibiting viral adhesion to the cell and by its viral anti-replicative effect, decreasing the viral load of patients with COVID-19, which may reflect in clinical improvement and better outcomes for these patients.

2. OBJECTIVES

2.1 Primary objective

Decay rate of the SARS-CoV-2 viral load logarithmic curve obtained via nasopharyngeal swab between the baseline and the fifth and tenth days after randomization.

2.2 Secondary objective

Evaluate the effect of halofuginone on:

I. Hospital admission between randomization and the day 28;

II. Need for invasive mechanical ventilation between randomization and day 28;

III. Time to resolve symptoms until the tenth day;

IV. Ordinal clinical scale of symptoms on 14th (1. not hospitalized, without limitation of daily activities; 2. not hospitalized, with limitation of daily activities; 3. hospitalized, without the need for supplemental oxygen; 4. hospitalized, requiring supplemental oxygen; 5 hospitalized, requiring high-flow nasal oxygen therapy, non-invasive mechanical ventilation, or both; 6. hospitalized, requiring blood oxygenation through a membrane system, invasive mechanical ventilation, or both; 7. Death).

3. METHODS: RESEARCH SUBJECTS, INTERVENTIONS AND OUTCOMES

3.1 Study design

Multicenter clinical trial, phase 2, randomized, double-blind, placebo-controlled in 150 patients.

3.2 Study locations

The study will be conducted in 2-5 Brazilian hospitals.

3.3 Recruitment plan

Patients will be recruited from outpatient clinics and emergency rooms at participating institutions.

3.4 Eligibility Criteria

3.4.1 Inclusion Criteria

1. Adult patients (age ≥ 18 years);
2. COVID-19 diagnosis confirmed by:
    a. Detection of SARS-CoV-2 by RT-PCR, or
    b. Rapid genetic or antigen tests registered by ANVISA;
3. Mild or moderate symptoms without indication for hospitalization;
4. Symptoms started seven days ago or less;
5. Be able to access the study's online questionnaire.

3.4.2 Exclusion Criteria

1. Pregnant or lactating women;
2. Known allergy or hypersensitivity to the study drug;
3. Patients at high risk of bleeding, defined by:
    a. Previous Intracranial hemorrhage,
    b. Ischemic stroke in the last 3 months,
    c. Anatomical vascular alteration of the central nervous system known, such as aneurysms or arteriovenous malformations,
    d. Malignant neoplasm of the central nervous system known,
    e. Metastatic solid neoplasia,
    f. Significant closed head or facial trauma in the last 3 months (defined as any trauma that required medical evaluation or hospitalization),
    g. Known intracranial abnormalities not listed as absolute contraindications (e.g., benign intracranial tumor),
    h. Bleeding in the last 2 to 4 weeks (excluding menstrual bleeding),

i. Surgical procedure in the last 3 weeks,

j. Current use of full-dose anticoagulants (warfarin, enoxaparin or new anticoagulants) or dual antiplatelet therapy,

k. Thrombocytopenia (<100,000/mL) or INR (international standardized ratio) > 1.3;

4. Renal failure, defined as glomerular filtration rate (GFR) estimated by the formulas MDRD or CKD-EPI <30mL/min/1.73m^2;
5. Previous participation in the study;
6. History of liver disease (cirrhosis) reported by the patient or in medical records, presence of esophageal varices or presence of ascites;
7. Decompensated heart failure, defined by the presence of dyspnea attributed to cardiac cause, edema of the lower limbs, crackles on pulmonary auscultation or pathological jugular turgency.
8. Participation in other clinical trials with antivirals in COVID-19

3.5 Procedures for COVID-19 diagnosis

For inclusion in the study, patients must present a diagnosis of SARS-CoV-2 infection defined by a positive RT-PCR test performed by the study center itself or present a confirmatory examination by RT-PCR performed at another center. If the time the result is available at the center takes more than 24 hours and the patient has carried out rapid automated tests or commercially similar, or rapid Antigen, although they are diagnostic tools registered by ANVISA to confirm the presence of SARS-CoV2, these results will be accepted for inclusion in the study.

3.6 Procedures for verifying the eligibility criteria

After signing the Informed Consent, it will be collected serum creatinine, prothrombin time, platelet count (blood count) and rapid pregnancy tests (in the case of women of childbearing age) to confirm the eligibility criteria. If the patient has underwent part or all the tests in the last 3 days, the results may be considered to verify the eligibility.

3.7 Contraception in the study patients

Although data from animal studies show that the studied doses had no effects on reproduction and embryotoxicity, the study will offer free condoms (male and female, depending on the participant's choice) for all men and women of childbearing age, as well as use guidance and orientation to sexual abstention for a period of 30 days from the study's inclusion.

3.8 Study Visits

For the HALOS trial, there will be three in person visits, one at the research center (day 1 - baseline) and two home visits (day 5 and day 10), two follow-up visits via phone call (day 14 and day 28). The study visits and their particularities are described below:

3.8.1 Day 1 - baseline

- Eligibility Check;
- Informed Consent Process and signature of the Form;
- Randomization;
- Collect demographic and baseline data;
- Collection of laboratory tests: complete blood count (CBC), urea, creatinine, alkaline phosphatase, gamma-GT, aspartate aminotransferase, alanine aminotransferase, direct and indirect

bilirubin, C-reactive protein, prothrombin time with RNI, activated partial thromboplastin time, fibrinogen, , sodium and potassium;

• Collection of nasopharyngeal swabs for SARS-CoV-2;

• Collection of urine for pregnancy test (in the case of women of childbearing age);

• Dispensing and guiding the use of study medication.

3.8.2 Day 5 – At home

• Collection of nasopharyngeal swabs for SARS-CoV-2;

• Collection of blood tube for analysis of serum level of halofuginone.

3.8.3 Day 10 – At home

• Collection of laboratory tests: complete blood count, urea, creatinine, alkaline phosphatase, gamma-GT, aspartate aminotransferase, alanine aminotransferase, direct and indirect bilirubin, C-reactive protein, prothrombin time with RNI, activated partial thromboplastin time, fibrinogen, , sodium and potassium.

• Collection of nasopharyngeal swabs for SARS-CoV-2;

• Collection of blood tube for analysis of serum level of halofuginone;

• Withdrawal and medication accounting from the study.

3.8.4 Day 14 and day 28 - follow-up via phone call

• Telephone follow-up to collect clinical data.

• At the follow-up visit on the day 14, information on the patient's status will be requested on the ordinal symptom scale (1. not hospitalized, without limitation of daily activities; 2. not hospitalized,

with limitation of daily activities; 3. hospitalized, unnecessary of supplemental oxygen; 4. hospitalized, requiring supplemental oxygen; 5. hospitalized, requiring high-flow nasal oxygen therapy, non-invasive mechanical ventilation, or both; 6. hospitalized, requiring blood oxygenation through a membrane system (ECMO), invasive mechanical ventilation, or both; 7 Death), presence of symptoms (myalgia, fever, shortness of breath, cough, diarrhea, nausea, vomiting and headache) and presence of adverse events (bleeding or other adverse events or symptoms reported by the patient).• At the follow-up visit on the day 28, information on vital status, need for hospitalization, need for invasive mechanical ventilation (in case of hospitalization) and presence of adverse events (bleeding or other adverse events or symptoms reported by the patient) will be requested.

3.9 Allocation concealment and randomization

Patients will be randomized in the proportion of 1:1:1, for the control arms, halofuginone 1mg and halofuginone 0.5mg. The randomization list will be generated electronically by an independent statistician using a specific software. Randomization will be performed in blocks and will be stratified by age (<60 years and ≥ 60 years).

The allocation concealment of the randomization list will be maintained through an automated and centralized randomization system accessed via the internet, available 24 hours a day. Patients who do not meet the eligibility criteria to participate in the study should not be recruited and randomized, and this will be considered a violation of the protocol. Patients, principal investigators and local staff will be kept blind to the allocation; only the sponsor will be aware of the blinding codes. The assessors and statisticians responsible for the results will be kept blind in relation to the allocated treatments.

3.10 Interventions

3.10.1 Treatment

Patients will be randomized at a 1: 1: 1 ratio to receive halofuginone 0.5mg orally once daily, halofuginone 1mg orally once daily or placebo. There will be two preparations of the halofuginone solution for the study, one in the concentration of 0.5 mg / 5 mL and the other in the concentration of 1 mg / 5 mL, as well as a solution containing placebo, which will ensure that the same volume of solution is administered for patients in both arms (0.5 mg and 1 mg) of the intervention and placebo, which will make it impossible to identify the randomized group. Thus, regardless of the group, each patient will use 5 mL of the designated solution per day, for 10 days. In case of need for hospitalization, the patient will stop using the study medication.

The study medication (intervention and placebo) will be sent to the participating center in properly identified bottles. Once the patient is randomized, a person from the clinical trial team will be responsible for delivering the medication to the patient, as well as to provide guidelines for use.

3.10.2 Additional study interventions

Knowing that the most commonly reported adverse events with the use of halofuginone in phase I trials were nausea and vomiting after taking the medication, patients who present such symptoms will be advised to prophylactic use of an antiemetic (ondansetron 4mg orally) between 30 minutes and 1h taking the study medication. This use should be maintained as long as the patient is using the study medication. If the patient does not experience nausea or vomiting with the use of the study medication, he should not use the antiemetic. Ondansetron will be provided by the study free of charge to patients who are not allergic to it. In case of allergy to ondansetron, metoclopramide 10mg will be offered as an antiemetic alternative.

In addition, there will be no other mandatory intervention in the clinical study. Patients will receive standard treatment indicated by the care team, as well as specific

guidelines on the disease. Specific treatment for COVID-19 may include, but is not limited to:

- Antibiotic therapy for secondary infection;
- Medications to treat symptoms such as fever, pain and nausea;
- Antivirals if indicated by the patient's medical team;
- Other interventions, if indicated by the patient's medical team.

3.10.3 Treatment interruption

Treatment should be stopped in the following situations:

- Patient presents hospitalization; **OR**
- Patient who had validated antigen test result and later presented a negative RT-PRC result performed on first day of the participation; OR
- Patient who has clinical indication for use of full-dose anticoagulants; **OR**
- Presence of serious adverse events.

In the situation of treatment interruption due to negative RT-PCR test for SARSCoV-2, no additional clinical and laboratory data will be collected.

In other situations, the patient will be instructed to stop using the study medication and there will be no additional collection of laboratory tests. Clinical data collection via online questionnaire and clinical follow-up via phone call will be maintained.

3.10.4 Halofuginone and dosage justification

Halofuginone [7-bromo-6-chloro-3- [3- (3-hydroxy-2-piperidinyl) -2-oxopropyl] - 4 (3H) -quinazolinone] is an analogue of the febrifugin used and approved by the FDA and the Brazilian Ministry of Agriculture, Livestock and Supply for the prevention of

coccidiosis in birds and against protozoa in cattle.[10] The molecule has a piperidine ring that is essential for its activity.[17]

The selected doses of halofuginone for the present study were based on safety, tolerability, pharmacokinetic and pharmacodynamic data published by de Jonge et al,[12] which included 24 patients with a median age of 58 years (62.5% men) and with refractory solid neoplasia standard treatment.

In preclinical studies, oral halofuginone doses of 0.35mg/kg/day and 0.142mg/kg/day, which correspond to doses of 24.5mg/day and 9.94mg/day for a 70kg adult, were well tolerated for periods of 4 to 26 weeks. In rats, mortality was observed at doses of 6mg/kg/day (corresponding to 420mg to 70/kg) after 5 days of treatment. The bioavailability of oral halofuginone in rats is 51% and in pigs 72%. In animals, about 60% of the oral dose is eliminated unchanged in the faeces. The plasma half-life varies between 5 and 17 hours. In the dose escalation study in adult males, no serious adverse events were reported at doses of 0.5 mg. In 1.5mg, 2mg and 2.5mg dose increments, mild to moderate nausea and vomiting were reported, however these episodes were short-lived and resolved spontaneously.

In adults, doses of 1mg/day per day for two weeks, no dose limiting toxicity (DLT) was noted. At doses of 2mg/day, some patients experienced nausea and vomiting grade 2 with improvement after the use of prophylactic antiemetics. However, with doses of 3.5mg per day, two patients had DLT, despite the use of prophylactic antiemetics, defining that the maximum tolerated dose (MTD) was exceeded.

Halofuginone caused predominantly symptoms of gastrointestinal toxicity, manifested by nausea and vomiting that were chronologically related to the use of medication. Most patients experienced symptoms within 30 minutes to an hour after taking the medication. Fatigue was the second most common side effect. Hematological toxicity was not observed. Table 1 describes the toxicity events during the two-week treatment period:

Table 1. Toxicity in the first two-week cycle according to the common terminology of adverse events of the National Cancer Institute version 2.0[18] [a]

Dose mg/day	No. of patients	Leukopenia				Neutropenia				Thrombocytopenia				Nausea				Vomiting				Fatigue				DLT
		1	2	3	4	1	2	3	4	1	2	3	4	1	2	3	4	1	2	3	4	1	2	3	4	
**1**	4									1				3				2				3				1*
**2**	6													1	4			2	3			2	3			
**3.5**	3														1	1		1	1					2		2
**0.5**	5	1												1				2				1				
**0.5 2x**	7													2				1	1			3				2*

[a] Adapted from de Jong et al.
[b] Abbreviations: DLT, dose-limiting toxicity.
*** DLT for chronic administration**

Seven of the 24 patients in the study experienced bleeding during treatment with halofuginone. In preclinical studies, halofuginone inhibited the migration of endothelial cells and prevented their organization in networks similar to capillary anastomoses. *In vivo* studies, no thromboembolic events or bleeding were observed. Table 2 describes the bleeding episodes and doses for each patient.

Table 2. Bleeding episodes [a]

Patient	Cycle [b]	Dose	Description
**1**	3	2mg/day	Patient with extensive metastatic melanoma, had bleeding in brain metastasis unknown at the time.
**2**	4	2mg/day	Gastrointestinal bleeding from benign gastric ulcer.
**3**	Uninformed	1mg/day	Patient with carcinoma of undetermined site had epistaxis.
**4**	Uninformed	0.5mg/day	A patient with metastatic renal neoplasia presented bleeding in a currently unknown brain metastasis and died.
**5**	1	0.5mg/day	Patient had an increase in old hematoma.
**6**	5	0.5mg/2x day	Patient with metastatic renal neoplasia died due to massive liver bleeding due to liver metastasis.
**7**	2-4	0.5mg/2x day	A patient with metastatic melanoma had gastrointestinal bleeding requiring multiple transfusions.

[a] Adapted from de Jong et al.
[b] Each cycle corresponds to a period of two weeks.

It is worth mentioning that bleeding complications are of concern, which led to the exclusion of groups at high risk of bleeding from the present study (previous intracranial bleeding, ischemic stroke in the last 3 months, anatomical vascular alteration of the central nervous system known [as aneurysms or arteriovenous malformations], known malignant neoplasm of the central nervous system, metastatic solid neoplasm, significant closed or facial head trauma in the last 3 months [defined as any trauma that required medical evaluation or hospitalization], known intracranial abnormalities not listed as absolute contraindications [ex: benign intracranial tumor], bleeding in the last 2 to 4 weeks [excluding menstrual bleeding], surgical procedure in

the last 3 weeks, current use of full-dose anticoagulants [warfarin, enoxaparin or new anticoagulants] or dual antiplatelet and platelet therapy to [<100,000/mL] or ISR [international standardized ratio]> 1.3). It is reinforced that in patients who presented hemorrhagic phenomena, laboratory tests that evaluate coagulation (platelet count and levels of coagulation factors) were not altered, which reinforces the hypothesis that these hemorrhagic phenomena are primarily related to the presence of metastases in these patients.

Absorption of halofuginone after an oral dose was associated with a maximum peak of the drug at 3.4 ± 4.8 hours. About 4-12% of the halofuginone administered was excreted in the urine, mainly in the first 24 hours after administration. Table 3 contains data on the pharmacokinetics of halofuginone in the study doses.

Table 3. Pharmacokinetics of halofuginone during the first cycle [a]

Dose (mg/day)	C_{max} (ng/mL)	T_{max} (h)	$T_{1/2}$ (h)
**0.5**	0.54	2.8	17.2
**1**	0.88	2.0	31.5

[a] Adapted from de Jong et al.
Abbreviations: C_{max}, plasma peak; T_{max}, time to maximum concentration; $T_{1/2}$, terminal disposal half life

Therefore, in view of the most common adverse events with the use of halofuginone (gastrointestinal symptoms such as nausea and vomiting, which were dependent on the dose administered, as well as duration of cycles), risk of bleeding, as well as pharmacokinetic properties of the drug, if by using doses of 1mg/day and 0.5mg/day in the intervention arms, excluding patients at high risk of bleeding, as well as patients who have less drug elimination.

3.10.5 Coding of study medication

Solutions with two different concentrations of halofuginone (0,5mg/5mL e 1 mg/5 mL) and a placebo solution will be used.

Each individual treatment solution will be identified by one of three random letters (from A to I) unique to each group. The code list will be sequential and unknown to researchers and patients, which the codes will be known exclusively by the pharmacist responsible for the study.

3.10.6 Emergency break of the designated treatment code

Emergency breaks in the code should be undertaken only when it is necessary to treat the participant safely. Very often, discontinuation of study treatment and knowledge of the possible designations of this treatment are sufficient to treat a participant who has an emergency condition.

Emergency breaks in the treatment code will be carried out using the study exclusive email and phone for this purpose.

When the investigator contacts the coordinating center to break a treatment code for a patient, he must provide the requested patient identification information and confirm the need to break the code for that patient. The investigator will then receive details of treatment with the investigational drug for the specified participant. An evaluation will be carried out by the team of the coordinating center and the designated doctor. After an emergency code break, the participant will follow the study in accordance with the protocol, but must discontinue the treatment of the study.

Figure 1. Emergency blinding break flowchart

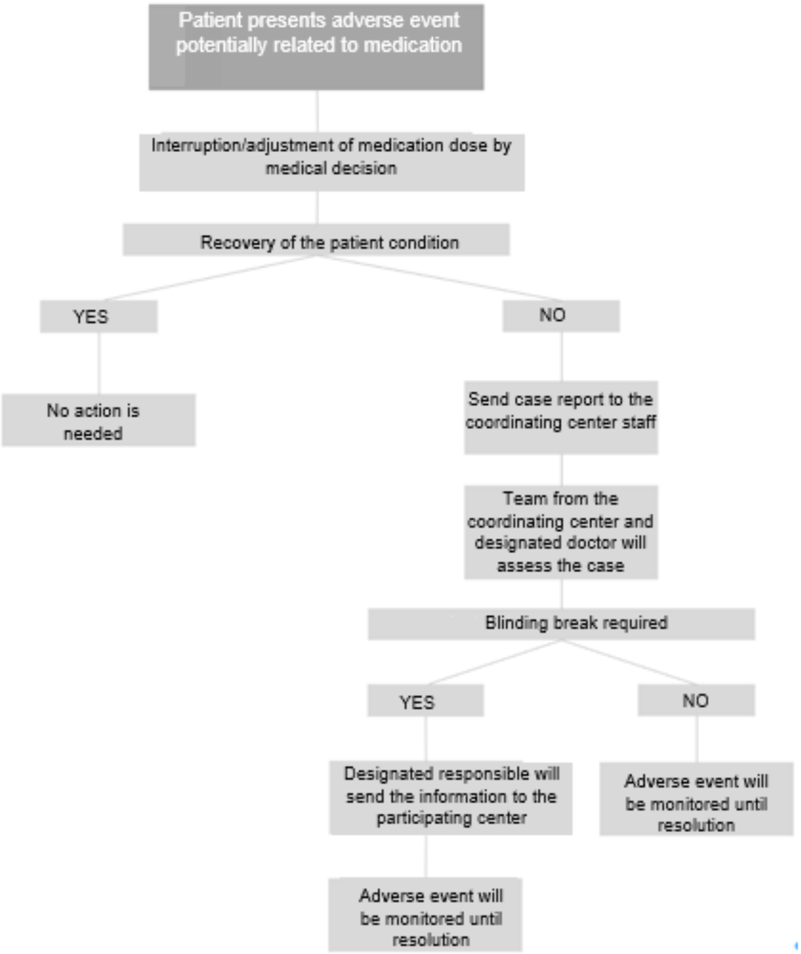

3.10.7 Adhesion

Adherence to the protocol will be guaranteed with the training of all investigators and study staff.

Once randomized, patients will receive guidance on the dosage of the study medication, mode of use and duration of treatment. Patients will also receive a newsletter that will contain clear guidelines on the use of the medication. During visits to collect exams on the fifth and tenth days, guidelines on dosage and use will be reinforced for the patient. On the tenth day, together with the collection of exams, the study medication bottles will be collected and sent to the pharmacist at the coordinating center, where the quantity of remaining medication will be measured, thus allowing inferences about the patients' adherence to treatment.

3.10.8 COVID-19 negative confirmatory exam

Patients who are included in the study based on the results of rapid tests such as GeneXpert Cepheid® (ANVISA Registration Number 81062710039) or commercially analogous, or rapid Antigen test (COVID - 19 Ag ECO Test - ANVISA Registration Number 80954880133) who have a negative laboratory diagnosis by RT-PCR for SARS-CoV-2 will be contacted via telephone where they will be informed to cease the use of the study medication. A study technician will go to the participant's residence to remove the study medication. The participant will be asked to allow the use of the data collected so far for the safety analysis of halofuginone, for analysis by intention to treat and other analyses.

3.10.9 Study Helpline

The study will have a helpline that will be available to answer urgent and non-urgent questions about the management of patients already recruited, as well as questions regarding the eligibility of potential patients for the study.

3.10.10 Losses to follow-up

The study basically consists of out-of-hospital monitoring of patients. Ultimately, obtaining outcome data depends on the contact of the study team with the patient. Strategies to avoid loss to follow-up such as obtaining multiple means of contact (e-mail, phone numbers, addresses) of the participants and an active search for exams and collection of medical records will be implemented.

In case the participant reports adverse events, the researcher in charge of the investigating center will be notified to contact the research participant, so that he is adequately monitored until the resolution of these events.

In situations which there is no success in contact with the patient, the last known clinical status of the patient will be assigned; sensitivity analyzes will be carried out disregarding patients with loss to follow-up in the analysis, and attributing the worst clinically plausible scenario to the losses, corresponding to the imputation in these

subjects of the results observed in the telephone follow-up of the group with the worst clinical results.

3.11 Collection, use and storage of biological material

3.11.1 Blood Tests

Collections of the following blood laboratory tests will be performed on the day of inclusion in the study (baseline) and on the tenth day: CBC, urea, creatinine, alkaline phosphatase, gamma-GT, aspartate aminotransferase, alanine aminotransferase, direct and indirect bilirubin, C-reactive protein , prothrombin time with INR, activated partial thromboplastin time, fibrinogen, sodium, potassium and a tube for serum analysis of the study drug (will also be collected on the fifth day). Baseline exams will be collected by trained staff from the participating center and 10th day exams will be collected by trained technical staff at the patient's home.

3.11.2 hCG Urine Test

The qualitative hCG Urine Test will be performed on baseline in women of childbearing age in order to verify the eligibility criteria

3.11.3 Nasopharyngeal swab

All patients in the study will perform nasopharyngeal swab collection to detect the SARS-CoV-2 viral load via RT-PCR on the day of inclusion in the study (baseline), and subsequently on the fifth and tenth days. The baseline swab will be collected by trained staff from the participating center and the exams on the fifth and tenth days will be collected by trained technical staff at the patient home.

Swabs for nasopharynx sample collection should be composed of rayon or dacron. A total of three swabs should be collected, one from each nasal orifice, scraping the meatus, and the other scraping from the palate to the pharynx. The three swabs

should be placed in bottles with 3mL of isotonic medium, preferably DMEM medium with 1% penicillin and streptomycin.

The samples can be stored in a refrigerator (from 2 to 8°C) for 24 hours, or for periods of months in a freezer –20°C or –70°C. As the shipment of the samples will occur within 24 hours, blue-ice can be used to guarantee refrigeration around 4°C.

3.11.4 Locals for processing, storage and performance of laboratory tests

The biological samples will be sent to the Central Laboratories daily. The Central Laboratories are the Clinical Laboratory of Hospital do Coração, DASA and the Laboratório de Investigações Médicas da Faculdade de Medicina da USP. The biological samples will be processed and analyzed at these laboratories. The routine tests will be performed at the DASA and at the Hospital do Coração; the serum dosage of Halofuginone will be performed at the Laboratório de Investigações Médicas da Faculdade de Medicina da USP and the assessment of SARS-CoV-2 viral load will be performed at Clinical Laboratory of Hospital do Coração. The storage of biological samples will be transient, for enough time to perform all the analyses, as necessary, and they will be destroyed according to Good Laboratory Practices.

All Central Laboratories have adequate infrastructure to develop this research protocol, as refrigerators with strict temperature control, restricted access and monitored by cameras 24 hours a day, 7 days a week. The staff involved on laboratory analyses is trained to perform its activities.

All biological material will be used exclusively for the purposes provided for in this protocol, in accordance with the provisions of Resolução CNS No. 441/2011 and Portaria No. 2.201/2011 and in compliance with the consent obtained from the research participants. There is no intention for future research using the biological samples collected in this study.

3.11.5 *Clinical data collection*

Data will be collected via a digital data collection form (e-CRF) accessed via the internet and developed by the IP-HCor data management team in conjunction with the researcher responsible for the study coordinating center and the responsible statistics team. This tool will be made available via a secure and specialized web platform to support data collection and management, in compliance with current regulations, in accordance with CFR 21 Part 11.

A health professional designated by the participating center will collect data on the day of inclusion of the study (baseline). All professionals who will participate in the study will undergo standardized training for data collection, through electronic conferences, communication via messages and 24 hours a day, every day of the week.

Research participants' data collected at Day 1 - baseline (Table 4)

- Demographic data: age, gender
- Baseline data: comorbidities, previous and current use of medications, date of onset of symptoms, clinical symptoms, date of positive SARS-CoV-2 test if available, clinical signs (temperature, heart rate, systolic and diastolic blood pressure, respiratory rate, peripheral oxygen saturation).

Table 4. Data from research participants and timeline

	Baseline/ D1	D2	D3	D4	D5	D6	D7	D8	D9	D10	D14	D28
**Screening**	X											
**Eligibility**	X											
**Informed Consent Form**	X											
**Randomization**	X											
**Interventions**												

Halofuginone 1mg/day	X	X	X	X	X	X	X	X	X	X		
Halofuginone 0.5mg/day	X	X	X	X	X	X	X	X	X	X		
Placebo	X	X	X	X	X	X	X	X	X	X		
**Data collection and tests**												
Demographic Data	X											
Baseline Data	X											
Symptoms and signs	X	X	X	X	X	X	X	X	X	X		
RT-PCR SARS-CoV-2	X				X					X		
Laboratory tests	X				X*					X		
Safety Data (via patients online questionnaire)	X	X	X	X	X	X	X	X	X	X		
Clinical and safety data via phone call											X	X

* Only serum level of halofuginone

Research participants data collected daily

Study participants will receive daily (until the tenth day) a secure link via email and / or phone messaging application to complete a questionnaire containing questions on clinical symptoms and use of study medication, and clinical signs (peripheral oxygen saturation and heart rate) that will be measured using a portable oximeter that will be provided to the patient free of charge by the coordinating center. This questionnaire will be stored in the same secure environment as the eCRF.

Follow-up calls will be made by the participating center team on the days 14 and 28. On the day 14 there will be data collection regarding the ordinal clinical scale of symptoms and on the day 28 the follow-up data: hospital admission, survival and use of mechanical ventilation.

4. OUTCOMES

4.1 Primary Outcome

Decay rate of the SARS-CoV-2 viral load logarithmic curve obtained via nasopharyngeal swab between the baseline and the fifth and tenth days after randomization.

4.2 Secondary Outcomes

I. Hospital admission between randomization and the day 28;
II. Need for invasive mechanical ventilation between randomization and day 28;
III. Time to resolve symptoms until the tenth day;
IV. Ordinal clinical scale of symptoms on day 14 (1. not hospitalized, without limitation of daily activities; 2. not hospitalized, with limitation of daily activities; 3. hospitalized, without the need for supplemental oxygen; 4. hospitalized, requiring supplemental oxygen; 5 hospitalized, requiring high-flow nasal oxygen therapy, non-invasive mechanical ventilation, or both; 6. hospitalized, requiring blood oxygenation through a membrane system, invasive mechanical ventilation, or both; 7. Death).

4.3 Secondary Safety Outcomes

I. Grade 1, 2, 3 and 4 adverse events, which were not present at the patient's entrance, defined by the *Division of AIDS* (DAIDS) *Table for Grading the Severity of Adult and Pediatric Adverse Events* (corrected version 2.1, July, 2017).
II. Bleeding, as defined in table 5:
    a. Major bleeding
    b. Clinically relevant non-major bleeding,
    c. Minor bleeding;
III. Adverse events (AE) and serious adverse events (SAE);
IV. Discontinuation of study drug-related treatment.

Table 5. Bleeding classification[19]

Type	Definition
**Major**	Defined as clinical bleeding associated with any of the following: fatal outcome, critical site involvement (intracranial, intraspinal, intraocular, pericardial, intra-articular, intramuscular with compartmental or retroperitoneal syndrome), or clinical bleeding with a drop in hemoglobin concentration ≥2g/dL, or need for transfusion of

	≥2 units of packed red blood cells or whole blood. All intracerebral (or intraparenchymal) bleeds are included in the primary analysis as hemorrhagic stroke.
**Clinically relevant non-major**	Defined as clinical bleeding that does not present major bleeding criteria, but requires medical intervention, unscheduled contact (in person or by telephone) with a doctor, temporary interruption of the study drug, pain or impairment of daily activities.
**Minor**	Defined as clinical bleeding that does not meet criteria for clinically relevant major or non-major bleeding.

5. SAMPLE SIZE

Based on data from the Wölfel et al. study (2020), an average linear decay rate for patients with SARS-CoV-2 in nasopharyngeal swab assessed from the fourth to the fourteenth day of symptom onset is 1.7 $\log_{10}$ every 5 days. Assuming this decay rate for the placebo group, considering the scenario that all selected active treatments will have a decay rate of 2.2 $\log_{10}$ (viral load), 150 patients with SARS-CoV-2 confirmed by RT-PCR (50 per group) will have 90% power to indicate that at least one of the treatments will be superior to the placebo, considering a two-tailed significance level of 2.7%, to guarantee an overall level of significance of 5%. The sample calculation was performed using simulations considering different standard deviations in each time step (baseline, 5 days and 10 days), and an auto-regressive correlation of 0.85. The covariance matrix was based on unpublished data on the daily viral load of Dr. Thiago Moreno Souza (Fiocruz).

6. STATISTICAL ANALYSIS

6.1 Outcomes

The analyzes will follow the modified intention to treat principle, considering only patients with laboratory diagnosis by positive RT-PCR for SARS-CoV-2.

The primary outcome will be a comparison of the viral load logarithm decay rates via RT-PCR to SARS-CoV-2 in 10 days between treatment groups compared to control. A linear list model will be used, assuming random intercept and slope for patients. But viral load measurements will be performed 5 and 10 days after randomization.

If the RT-PCR exam is not performed for any reason, imputation will be performed using only an estimate via the chain equation method using the mice package. We chose to perform the primary outcome assessment based on multiple imputation methods to correct any viral load values due to loss of information due to mortality, however, based on data by Cavalcanti et al. (2020), the expected mortality during the period of exam collection is minimal (<3%).

6.2 Safety interim analysis

A safety interim analysis is planned after 60 patients have reached the outcome of the 28-day follow-up study. The Data Monitoring Committee (DMC) will use the Haybittle-Peto rule, considering p <0.001, to interrupt the study for safety reasons. In view of the outcome of the study and sample size, the interim analysis will not include an analysis of effectiveness. In this analysis, the DMC will also consider safety issues such as the number of adverse events between the arms to make a decision. The Haybittle-Peto rule will be used as a reference, not as a strict rule. DMC will also consider the effect on other secondary outcomes, in particular on adverse events, and on new external evidence available during the conduct of the clinical study. We will not adjust the final hypothesis test values for sequential analysis

7. DATA QUALITY CONTROL

7.1 Data quality assurance

The quality and inconsistencies of the data will be periodically assessed by the IP-HCor data management team. Inconsistencies will be reported to the centers for possible reassessments and corrections. Once it is identified that a center has shown a high frequency of inconsistencies, monitoring will be carried out to verify the information in the source documents and new training of the team to collect and insert the data into the system.

Several procedures are planned to guarantee the quality and traceability of the data inserted in the system, including, but not limited to:

- All researchers will participate in a training session before the start of the study to ensure consistency of the study procedures, including data collection;
- Investigators will be able to call the Coordinating Center study to resolve issues or problems that may arise;
- Data cleaning to identify inconsistencies will be conducted periodically (every fifteen days). The centers will be notified of inconsistencies in order to provide correction;
- The IP-HCor data management team will review detailed reports on screening, inclusion, follow-up, consistencies and completeness of the data on a monthly basis.
- Immediate actions will be taken to resolve any problems.

Monitoring in the participating centers will be carried out during the conduct of the study in particular if there are special needs due to the accumulation of inconsistencies or missing data.

8. ETHICAL ASPECTS AND GOOD CLINICAL PRACTICE

The study will be conducted in accordance with current national regulations, described in the following documents:

- Resolução CNS/MS No. 466, of December 12, 2012 and its complementaries from CNS/MS;
- Resolução CNS/MS No. 441, of May 12, 2011 and Portaria No. 2.201, of September 14, 2011.
- Document of the Americas.

8.1 Study Approval

The ethical approval will be obtained according to all applicable regulatory obligations. All the ethical and regulatory approvals must be available before any

procedure involving the research subject, including the screening tests to verify ones elegibility,

Considering the provisions of the CONEP guidance letter “Guidelines for conducting CEP research and activities during the pandemic caused by the SARS-COV-2 coronavirus (COVID-19)”, dated May 9, 2020, item 2.1.2, where it says: “In the case of research protocols that have “participating centers” and / or “co-participating centers”, no new ethical analysis should be carried out by the respective linked CEPs. Due to the exceptional character adopted, CEPs will endorse the approved opinion, when applicable, issued by CONEP”. The participating centers must wait for a referenced opinion from their institution's CEP to start the study at the institution.

In addition, protocol amendments should be implemented only after due approval by the CEP/CONEP System, following the orientations described above, except in situations where the implementation of the amendment prior to the approval of the CEP/CONEP system aims to protect research participants. In this case, the CEP/CONEP system must be notified of this implementation.

8.2 Informed Consent Process

Each research participant or their legal representative must provide their written consent in accordance with local requirements, after the nature of the study has been fully explained. The ICF(s) must be signed before any activity related to the study is carried out. The ICF(s) that are used must be approved by the coordinating center and by the REC of the participating centers, and must be in a language that the research participant is able to read and understand. Informed consent must comply with the principles originated in Resolução 466/12 of the National Health Council and with the guidelines of Good Clinical Practices.

Prior to inclusion in the study, the investigator or an authorized member of the research center team must explain to potential research participants or their legal representative the purposes, methods, reasonably anticipated benefits and potential risks of the study, as well as any discomfort that participation in the study may cause. Research participants or their legal representatives will be informed that their

participation is voluntary and that they will be able to withdraw their consent to participate at any time. They will be informed that the choice not to participate will not affect the care they will receive to treat their illness.

Finally, they will be informed that the investigator will keep a record of identification of the research participants and that their records will be accessible by health authorities and the authorized team of the coordinating center without violation of the research participant’s confidentiality, at the level permitted by applicable law(s) or regulation(s).

The research participant or his legal representative will have enough time to read the ICF and will have the opportunity to ask questions. After this explanation and before entering the study, consent must be properly registered through the dated personal signature of the research participant or his legal representative and whoever explained it. After obtaining consent, a copy of the ICF must be provided to the research participant. This entire process must be documented in the participant’s source document.

8.3 Criteria for discontinuing study participation

The patient will be withdrew from the study if he withdraws his consent to participate in the study. Details of participant withdrawal must be documented in the source document and eCRF. Upon withdrawal from the study, drug treatments will be determined by the patient's assistant physician. The patient may withdraw his consent to participate in the study for any reason and at any time, without prejudice to the continuity of his medical care.

The request to withdraw consent means that the patient no longer wants to participate in the study, that is, does not want to be evaluated and monitored in the study, perform any procedures related to the study, or be contacted again by the research team. Furthermore, further analysis of previously collected biological materials will not be allowed. In this situation, the study team will only contact the participant to withdraw the study medication. Local investigators must document withdrawal of patient consent and inform the Coordinating Center. Patients who withdraw consent

and discontinue their participation in the study after randomization will not be replaced by other participants.

Randomized patients may also refuse to participate in specific aspects of the study or refuse to continue using the proposed drugs, without withdrawing their consent to participate. In this case, patients should be asked for permission to continue to follow them until the end of the study. Local investigators must make every effort to accommodate the needs of patients to maintain their participation in the study.

It is reinforced that only patients with a positive diagnosis of COVID-19 will be included in the study. However, after randomization (D1), the RT-PCR test will be collected from all participants, necessary for standardizing the test and collection to obtain the viral load of SARS-CoV-2 on the day of inclusion in the study. Although the false-negative rates for rapid tests and RT-PCR that confirmed the diagnosis of COVID-19 in participants (prior to inclusion) are low, it is possible that the participant will have a positive test at the time of inclusion and a negative test. in the first collection of the study (D0). In this circumstance, the participant will no longer receive the proposed treatment, and additional clinical and laboratory data will not be collected.

8.4 Risks and discomforts

There is a risk of breach of confidentiality of information collected from research participants. This risk is inherent in any research, and is not greater in the present research. To mitigate these risks, the records of participation in this study will be kept confidential and will be accessed in a restricted way only by people linked to the study, who will transfer the clinical information to specific forms (which do not have information that can identify the research participant) and verify that the study is being carried out properly. Only a number generated at the beginning of the study, the initials and / or date of birth will be used to identify the participant. The confidentiality and privacy of all information will be ensured.

There may still be some risk of embarrassment, however, the study team is trained to approach the participant in a way that does not cause any malaise or discomfort of this type, always thinking about the participant's well-being and freedom.

The electronic data collection form will identify the patient and the investigating center by the corresponding number. The data obtained from the medical record must be kept confidential by the investigator centers, in lockers with restricted access and the guarantee of anonymity of all data in provisional and definitive reports will be ensured.

Other potential risks to the research participant are related to the collection of biological samples. Regarding blood collections, these do not differ from collections for routine exams. Thus, there may be local pain, bruising or phlebitis. Collections of nasopharyngeal swab samples may also incur discomfort, which is transient and of moderate intensity. In order to mitigate risks inherent to these procedures, the collections will be carried out by a trained health professional, as designated by the research center (for the baseline exams) and by a trained team designated by the coordinating center to collect the other exams.

There are potential risks related to the use of halofuginone. The main risks are gastrointestinal symptoms such as nausea and vomiting, as well as fatigue. Regarding nausea and vomiting, antiemetics will be prescribed and provided free of charge to patients, following the guidelines of the protocol. Another potential risk of the use of halofuginone refers to a greater chance of bleeding, however, this risk is mitigated by clear and specific criteria and exclusion, not allowing the inclusion of patients with a high risk of bleeding in the study.

8.5 Benefits for Participants

Currently, the benefits of treating SARS-CoV-2 with halofuginone are potential, but unknown. Due to its mechanism of action, halofuginone has the potential to be effective in treating patients with COVID-19, decreasing viral replication and potentially preventing severe forms of the disease. If it proves to be effective, treatment can contribute to better clinical outcomes of the disease in patients in the intervention group.

Research participants will receive the results of laboratory tests collected by the research protocol via specific link protected by password, individual and secure that will be sent via email and telephone message.

8.6 Reporting adverse events

8.6.1 Adverse Events (AE)

An adverse event (AE) is any unfavorable medical occurrence in a clinical study participant who has been administered a drug and who does not necessarily have a causal relationship to treatment. An AE can, therefore, be any unfavorable and/or unintended sign, symptom or disease, temporarily associated with the use of a study drug, and whether or not considered to it. AEs may also include pre- or post-treatment complications that occur as a result of procedures specified in the protocol, lack of efficacy, overdose, reports of drug abuse/misuse or professional exposure. Pre-existing events that increase in severity or change in nature during or as a result of participating in the clinical study will also be considered AEs.

An AE does not include the following:

- Medical or surgical procedures, such as surgery, endoscopy, tooth extraction and transfusion. The condition that led to the procedure can be an adverse event and must be reported.
- Pre-existing diseases, conditions or laboratory abnormalities present or detected before entering the study that do not get worse
- Situations in which an undesirable medical event has not occurred (for example, hospitalization for elective surgery, social and/or convenience admissions)
- Overdose without clinical sequelae
- Any medical condition or clinically significant laboratory abnormality with a start date prior to the date of signing the consent form, and not related to a procedure associated with the protocol. It is considered pre-existing and must be documented in the medical history and CRF

All adverse events must be recorded by the investigators on the clinical records (CRF), regardless of severity.

8.6.2 Serious Adverse Events (SAE)

All serious adverse events (SAE) should be recorded by the investigators on the clinical records (CRF) within a maximum of 24 hours from the moment the investigator became aware of the event. Serious adverse event is the one that results in any adverse experience with drugs, biological products or devices, occurring at any dose and that results in any of the following outcomes:

a) death;

b) threat to life;

c) persistent or significant incapacity/disability;

d) requires hospitalization or prolongs hospitalization;

e) congenital anomaly or birth defect;

f) any suspicion of transmission of an infectious agent by means of a drug or;

g) clinically significant event.

The principal investigator at the research center is responsible for informing research ethics committees of any serious adverse events as required by local regulations within 24 hours.

The HCor Research Institute is responsible for receiving and monitoring all adverse events that occurred during the clinical study. All adverse events classified as serious according to the definition of RDC 09/15 must be informed to the sponsors within a maximum period of 24 hours through the specific email and the Serious Adverse Event Notification Form, according to RDC 09/15.

The causal realtionship of all adverse events (serious and non-serious) with the products under investigation (Halofuginone or placebo) is characterized as follows:

- Related: There is clear evidence that the event is related to the use of the products under investigation.
- Possible: The event cannot be explained by the clinical condition of the research participant, concomitant therapy, or other causes, and there is a plausible temporal relationship between the event and the administration of the products under investigation.

- Improbable An event for which an alternative explanation is more likely (e.g., concomitant medications or ongoing clinical conditions), or the temporal relationship to exposure to products under investigation suggests that a causal relationship is unlikely.
- Not Related: The event can be explained immediately by the research participant's underlying clinical condition, concomitant therapy, or other causes.

Data regarding security outcomes of interest will be collected as described in section **4.3**.

8.6.3 Direct or indirect pregnancy

The direct or indirect pregnancy must be registered by investigators on the eCRF within 24 hours after staff knowledge.

In both cases the follow-up will be performed via phone call by staff site team in 60 and 90 days, additionally to the planned 28 days follow-up.

8.7 Confidentiality

This study will be conducted by the clinical team and those involved in the study will guarantee the maintenance of the privacy and confidentiality of each study participant. Participants will not be identified in any published reports of this study. All records will be kept confidential to the extent provided by international and local law. No information about the study or data will be disclosed to unauthorized third parties.

It is likely that the stored research data will have significant value in the future for other studies and, therefore, the grouped database study may be shared, as provided in the ICF. No information that allows identifying research participants will be shared.

Paper and electronic medical records can be accessed during the study to confirm, verify or complete the clinical information provided on the case report form.

The center files will always be accessible only to the clinical and research staff. Researchers consent will be requested to access patient data. The local research team will access personal information, but all data will be anonymized before the transfer.

9. AUXILIARY STUDIES

No auxiliary studies are planned.

10. DATABASE LOCK AND CLINICAL STUDY SUPERVISION

The database will be locked after the primary study completion date (obtaining the outcome data within 28 days of the last patient included), together with all necessary actions to obtain follow-up. Access to the database will be granted to members of the steering committee and statisticians before the publication of the main results. Limited access to blocked data can be granted by the steering committee to CoviCept Therapeutics, Inc. before the results are published.

The study completion date, defined as "the date on which the last participant in a clinical study was examined or received an intervention/treatment for the collection of final data for the measurement of the primary outcome", is the telephone contact for obtaining clinical outcomes and 28-day safety of the last patient included.

11. STUDY ORGANIZATION

11.1 Study coordination

The study will be coordinated by IP-HCor. The coordinating center teams will provide training, guidance and support to participating centers to ensure adherence to the data collection protocol. The team has the experience and level of knowledge in research methods and necessary biostatistics.

The Coordinating Center responsibilities include:

- Planning and conducting the study;
- Protocol review;

- Preparation of electronic data collection forms (CRFs);
- Preparation of operations manual;
- Data quality management and control: Development, testing and maintenance of the electronic data capture system; Continuous control of data quality;
- Selection and training of participating centers;
- Assist centers in preparing regulatory dossiers for submission to IRB;
- Distribution of study medications to the centers
- Monitoring recruitment rates
- Monitor follow-up and implement actions to prevent loss to follow-up;
- Coordinate the collection of laboratory tests and outcome data;
- Development of auxiliary material for the study;
- Complete statistical analysis;
- Assist in the preparation of the final manuscript.

11.2 Clinical study monitoring

The Steering Committee is responsible for the general supervision of the study, assisting in the development of the study protocol and in the preparation of the final manuscript. All other study committees report to the Steering Committee. Steering Committee members are researchers trained in the design and conduct of randomized clinical trials. Steering Committee members will work to ensure proper inclusion in the clinical trial through daily contact with recruitment centers, and through webinars, in which centers can participate and discuss possible issues.

The Data Monitoring Committee (DMC) is made up of a statistician and two doctors independent of the study researchers. The DMC is responsible for advising the Steering Committee on the continuity of the study as planned or interrupting recruitment based on the evidence that the intervention of the experimental group results in increased mortality or adverse events compared to the control. At the beginning of its activities, the DMC will prepare a booklet specifying the details of the formation of the DMC, its operation, meetings and rules for interruption. The DMC will

also receive a report of the events. After considering the preliminary analysis, adverse events and, occasionally, external evidence, the Data Monitoring Committee should assess whether there is evidence beyond reasonable doubt that the intervention is clearly contraindicated for all patients.

11.3 Interruption of clinical study

The DMC is responsible for advising the Steering Committee on the continuity of the study as planned or interrupting recruitment based on the evidence that the intervention of the experimental group results in increased mortality or adverse events compared to the control. There is a safety analysis planned by the DMC immediately after the first 60 patients have 28-day outcome data obtained. According to the DMC framework, and based on the continuous receipt of adverse events, and occasional external evidence, the DMC may recommend discontinuing study treatment after the planned preliminary analysis or at any time if there is evidence beyond doubt reason that the intervention is clearly dangerous for patients.

12. DISSEMINATION OF RESULTS

The Sponsor/Coordinating Center (HCor Research Institute) may freely publish and distribute the results of this study or, otherwise, publish or submit for publication of an article, manuscript, abstract, report, poster, presentation or other material containing or addressing the results of this study. The Sponsor/Main Investigator must send CoviCept Therapeutics, Inc. a copy of any proposed publication thirty days prior to submission for Publication. CoviCept Therapeutics, Inc. may make comments, but may not make any editorial changes to the proposed publication.

13. ADMINISTRATIVE ASPECTS

13.1 The role of COVICEPT Therapeutics

The team of the coordinating/sponsoring center has independent control over the study and is the only party responsible for conducting it. Consequently, he also assumes responsibility as a sponsor of the study in accordance with the regulations.

Therefore, the role of CoviCept Therapeutics, Inc. is limited to the following:

1. CoviCept Therapeutics, Inc. will supply the team of the coordinating/sponsoring center with the drug halofuginone for the preparation by the coordinating center of the medication solutions in the active treatment arms;
2. CoviCept Therapeutics, Inc. will disclose, to the team of the coordinating/sponsoring center, all the necessary confidential information about the halofuginone study drug to allow its safe use;
3. CoviCept Therapeutics, Inc. will provide additional financial support to the coordinating/sponsoring center team, in accordance with fair market value practices
4. CoviCept Therapeutics, Inc. will not have access to data that can identify individual patients, nor will it be involved in the decision to publish the results.

13.2 Administrative and legal obligations

Investigators will ensure compliance with all requirements of the International Council's Good Clinical Practice Directive on Harmonization of Technical Requirements in Pharmaceutical Products for Human Use (ICH) while conducting this study.

13.3 Status of sanitary regularization of the Product Under Investigation

The product under investigation Halofuginone is not regularized before the National Health Surveillance Agency (ANVISA). This study does not intend to subsidize the regularization of the product before this regulatory agency.

13.4 Diverse

It is important to note that, in view of the magnitude of the pandemic, the performance of serial clinical studies (beginning of one study after the completion of another) in several countries leads to a delay in the generation of evidence, thus opting for studies in parallel (with the possibility of carrying out similar studies in other populations).

The present study is an initiative of the researcher, which partially justifies the accomplishment of it in national territory. Despite, such medication may be evaluated in the future for the treatment of other viral diseases endemic in our country, such as dengue, zika virus and chikungunya.

14. REFERENCES

1. World Health Organization. WHO Director-General's opening remarks at the media briefing on COVID-19 - 11 March 2020: WHO; 2020 [Available from: https://www.who.int/dg/speeches/detail/who-director-general-s-opening-remarks-at-the-media-briefing-on-covid-19---11-march-2020 accessed March 25th 2020.
2. Group RC, Horby P, Lim WS, et al. Dexamethasone in Hospitalized Patients with Covid-19 - Preliminary Report. *N Engl J Med* 2020 doi: 10.1056/NEJMoa2021436 [published Online First: 2020/07/18]
3. Huang Y, Yang C, Xu XF, et al. Structural and functional properties of SARS-CoV-2 spike protein: potential antivirus drug development for COVID-19. *Acta Pharmacol Sin* 2020;41(9):1141-49. doi: 10.1038/s41401-020-0485-4 [published Online First: 2020/08/05]
4. Cevik M, Kuppalli K, Kindrachuk J, et al. Virology, transmission, and pathogenesis of SARS-CoV-2. *BMJ* 2020;371:m3862. doi: 10.1136/bmj.m3862 [published Online First: 2020/10/25]
5. Shang J, Ye G, Shi K, et al. Structural basis of receptor recognition by SARS-CoV-2. *Nature* 2020;581(7807):221-24. doi: 10.1038/s41586-020-2179-y [published Online First: 2020/04/01]
6. Clausen TM, Sandoval DR, Spliid CB, et al. SARS-CoV-2 Infection Depends on Cellular Heparan Sulfate and ACE2. *Cell* 2020;183(4):1043-57 e15. doi: 10.1016/j.cell.2020.09.033 [published Online First: 2020/09/25]
7. Cevik M, Bamford CGG, Ho A. COVID-19 pandemic-a focused review for clinicians. *Clin Microbiol Infect* 2020;26(7):842-47. doi: 10.1016/j.cmi.2020.04.023 [published Online First: 2020/04/29]
8. Dai L, Gao GF. Viral targets for vaccines against COVID-19. *Nat Rev Immunol* 2020 doi: 10.1038/s41577-020-00480-0 [published Online First: 2020/12/20]
9. Keech C, Albert G, Cho I, et al. Phase 1-2 Trial of a SARS-CoV-2 Recombinant Spike Protein Nanoparticle Vaccine. *N Engl J Med* 2020;383(24):2320-32. doi: 10.1056/NEJMoa2026920 [published Online First: 2020/09/03]
10. Pines M, Spector I. Halofuginone - the multifaceted molecule. *Molecules* 2015;20(1):573-94. doi: 10.3390/molecules20010573 [published Online First: 2015/01/09]
11. Keller TL, Zocco D, Sundrud MS, et al. Halofuginone and other febrifugine derivatives inhibit prolyl-tRNA synthetase. *Nat Chem Biol* 2012;8(3):311-7. doi: 10.1038/nchembio.790 [published Online First: 2012/02/14]
12. de Jonge MJ, Dumez H, Verweij J, et al. Phase I and pharmacokinetic study of halofuginone, an oral quinazolinone derivative in patients with advanced solid tumours. *Eur J Cancer* 2006;42(12):1768-74. doi: 10.1016/j.ejca.2005.12.027 [published Online First: 2006/07/04]
13. Koon HB, Fingleton B, Lee JY, et al. Phase II AIDS Malignancy Consortium trial of topical halofuginone in AIDS-related Kaposi sarcoma. *J Acquir Immune Defic Syndr* 2011;56(1):64-8. doi: 10.1097/QAI.0b013e3181fc0141 [published Online First: 2010/11/12]

14. Nativ O, Thorpe A, Laufer M, et al. Safety and tolerability of oral halofuginone hydrobromide in refractory recurrent transitional cell carcinoma of the bladder. *Journal of Clinical Oncology* 2004;22(14_suppl):4757-57. doi: 10.1200/jco.2004.22.90140.4757
15. Jain V, Yogavel M, Oshima Y, et al. Structure of Prolyl-tRNA Synthetase-Halofuginone Complex Provides Basis for Development of Drugs against Malaria and Toxoplasmosis. *Structure* 2015;23(5):819-29. doi: 10.1016/j.str.2015.02.011 [published Online First: 2015/03/31]
16. Stecklair KP, Hamburger DR, Egorin MJ, et al. Pharmacokinetics and tissue distribution of halofuginone (NSC 713205) in CD2F1 mice and Fischer 344 rats. *Cancer Chemother Pharmacol* 2001;48(5):375-82. doi: 10.1007/s002800100367 [published Online First: 2002/01/05]
17. Kamberov YG, Kim J, Mazitschek R, et al. Microarray profiling reveals the integrated stress response is activated by halofuginone in mammary epithelial cells. *BMC Res Notes* 2011;4:381. doi: 10.1186/1756-0500-4-381 [published Online First: 2011/10/07]
18. Saad ED, Hoff PM, Carnelós RP, et al. Critérios Comuns de Toxicidade do Instituto Nacional de Câncer dos Estados Unidos. *Revista Brasileira de Cancerologia* 2002;40(1):63-96.
19. Schulman S, Kearon C. Definition of major bleeding in clinical investigations of antihemostatic medicinal products in non-surgical patients. *J Thromb Haemost* 2005;3(4):692-4. doi: 10.1111/j.1538-7836.2005.01204.x [published Online First: 2005/04/22]
