# Supplementary material for: Halofuginone for non-hospitalized adult patients with COVID-19 a multicenter, randomized placebo-controlled phase 2 trial. The HALOS trial
Source: PLoS One. 2024 Feb 23;19(2):e0299197. doi: 10.1371/journal.pone.0299197 (PMC10889621; doi:10.1371/journal.pone.0299197)
Supplement: S3 Appendix — (DOCX) [file pone.0299197.s003.docx]

**S1 Appendix**

**Methods**

**Additional information on the study drug**

Halofuginone is an analogue of the febrifugin (an alkaloid derived from *Dichroa febrifuga*, one of the 50 fundamental herbs of traditional Chinese medicine)^1,2^ used and approved by the FDA and the Brazilian Ministry of Agriculture, Livestock and Supply for the prevention of coccidiosis in birds and against protozoa in cattle.^1^ The molecule has a piperidine ring that is essential for its activity.^3^

Through screening of epigenetic and translational regulatory compounds, halofuginone has been identified as a potent inhibitor of SARS-CoV-2 cell adhesion dependent on protein S and heparan sulfate (HS).^4^ In cultures of human bronchial epithelium infected with SARS-CoV-2, halofuginone in concentrations of 10nM and 100nM significantly reduced the number of cells infected by SARS-CoV-2 without affecting cell viability.^4^

Halofuginone is known to be a potent inhibitor of the prolyl-tRNA synthetase (PRS).^2^ Inhibition of PRS suppresses the translation of proline-rich proteins, while having a minimal effect on general protein synthesis.^2^ It was demonstrated that halofuginone inhibits SARS-CoV-2 viral replication, showing a reduction of about 1000 times in the number of virions secreted in cells treated with halofuginone, as well as a drop in mRNA intracellular protein S of SARS-CoV-2 in more than 20,000 times, with an average inhibitory concentration (IC50) of 34.9nM.^4^

Commercial halofuginone is a racemic mixture of dextrogyrous and levogyrous enantiomers, ^2,4,5^ and was synthesized under GMP conditions (Nanosyn, Inc, Santa Clara, CA, USA) with slight modification of a prior method.^6^ It was observed that the enantiomer active in the viral inhibition of SARS-CoV-2 is the dextrogyrus, with an IC50 of 12nM. Oral halofuginone has high bioavailability, with a maximum serum concentration (Cmax) of 0.54ng/mL (~1.3nM) after oral 0.5mg dose and Cmax 3.09ng/mL (~7.4nM) after oral 3.5mg dose in a phase I study^7^ and data show that the concentration of halofuginone in lung tissue can be up to 87 times higher than in plasma.^8^

Placebo and both halofuginone solutions were prepared in identical bootless, with only distinction being the randomization group letter. All bottles were prepared with 60ml of diluent (aqueous solution of 10% ethanol + 0.01N Hydrochloric acid), with concentration of halofuginone solutions of 0.5mg/5ml and 1mg/5ml. All final solutions (placebo and halofuginone) had same visual aspect with no difference in color, taste or smell and were refrigerated at 4^o^C for the duration of the treatment period. Patients were instructed to take orally 5ml of the designed solution (measured with a syringe provided by the study) daily from day 1 to day 10.

**Additional information on inclusion and exclusion criteria**

We enrolled patients who presented at the emergency department of the participating centers. All patients with suspected Covid-19 diagnosis performed either a confirmatory exam of RT-PCR or rapid genetic or antigen tests. Patients who presented at the emergency department with already a positive confirmatory test for Covid-19 this test was accepted as a positive test for entering the study. All women in childbearing age were tested for pregnancy before entering the study.

- Each patient had to fulfill all the following inclusion criteria to be eligible for enrolment:

- Age ≥18 years old;
- Confirmed Covid-19 diagnostic by detection of SARS-CoV-2 by reverse transcription polymerase chain reaction (RT-PCR), rapid genetic test or antigen test;
- Mild or moderate symptoms without indication for hospitalization;
- Symptoms ‘onset seven days ago or less;
- Abillity to access the study's online questionnaire

- Exclusion criteria:

- Pregnancy or active lactation;
- Known allergy or hypersensitivity to the study drug;
- High risk of bleeding, defined by:
  - Previous intracranial hemorrhage,
  - Ischemic stroke in the past 3 months,
  - Known anatomical vascular malformation of the central nervous system, such as aneurysms or arteriovenous malformations,
  - Known malignant neoplasm of the central nervous system,
  - Metastatic solid neoplasia,
  - Significant closed head or facial trauma in the past 3 months (defined as any trauma that required medical evaluation or hospitalization),
  - Known intracranial abnormalities not listed as absolute contraindications (e.g., benign intracranial tumor),
  - Bleeding in the past 2 to 4 weeks (excluding menstrual bleeding),
  - Surgical procedure in the past 3 weeks,
  - Current use of full-dose anticoagulants (warfarin, enoxaparin or new anticoagulants) or dual antiplatelet therapy,
  - Thrombocytopenia (<100.000/mL) or INR (international standardized ratio) > 1.3;
- Renal failure, defined as glomerular filtration rate (GFR) estimated by the formulas MDRD or CKD-EPI <30mL/min/1.73m^2^;
- Previous participation in the study;
- History of liver disease (cirrhosis) reported by the patient or in medical records, presence of esophageal varices or ascites;
- Decompensated heart failure, defined by the presence of dyspnea attributed to a cardiac cause, lower limbs edema, rales on pulmonary auscultation or pathological jugular distension;
- Participation in other clinical trials with antivirals in COVID-19

**Additional information on study medication adherence**

Information on drug adherence was collected daily using the online questionnaire send to all study patients. Patients were instructed to respond if they took the study medication and if not, the reason for not taking it. For the per protocol analysis, we considered all patients that took at least 8 doses of halofuginone.

**Additional information on symptoms-free days**

We reported the secondary outcome of time to resolve symptoms until the tenth day a symptoms-free days up to day 10. This decision was made due to the possibility of a patient developing new symptoms after the inclusion, which the outcome of time to resolve symptoms until the tenth day would not capture. One symptoms-free day is defined by the absence of all following clinical symptoms: cough, dyspnea, rhinorrhea, nausea vomit, fever, muscle or joint pain, headache, and fatigue.

**Additional information adverse events**

All adverse events were evaluated using the patient’s online questionnaire and data collected on the 14^th^ and 28^th^ day telephone follow up call. Any symptoms reported by the patient which was not present at study’s entry was considered an adverse event.

**References**

1. Pines M, Spector I. Halofuginone - the multifaceted molecule. *Molecules*. Jan 5 2015;20(1):573-94. doi:10.3390/molecules20010573

2. Keller TL, Zocco D, Sundrud MS, et al. Halofuginone and other febrifugine derivatives inhibit prolyl-tRNA synthetase. *Nat Chem Biol*. Feb 12 2012;8(3):311-7. doi:10.1038/nchembio.790

3. Kamberov YG, Kim J, Mazitschek R, Kuo WP, Whitman M. Microarray profiling reveals the integrated stress response is activated by halofuginone in mammary epithelial cells. *BMC Res Notes*. Oct 5 2011;4:381. doi:10.1186/1756-0500-4-381

4. Sandoval DR, Clausen TM, Nora C, et al. The Prolyl-tRNA Synthetase Inhibitor Halofuginone Inhibits SARS-CoV-2 Infection. *bioRxiv*. Mar 26 2021;doi:10.1101/2021.03.22.436522

5. Jain V, Yogavel M, Oshima Y, et al. Structure of Prolyl-tRNA Synthetase-Halofuginone Complex Provides Basis for Development of Drugs against Malaria and Toxoplasmosis. *Structure*. May 5 2015;23(5):819-829. doi:10.1016/j.str.2015.02.011

6. Xu H, Yin W, Liang H, Nan Y, Qiu F, Jin Y. A Scalable Total Synthesis of Halofuginone. *Organic Process Research & Development*. 2019/05/17 2019;23(5):990-997. doi:10.1021/acs.oprd.9b00059

7. de Jonge MJ, Dumez H, Verweij J, et al. Phase I and pharmacokinetic study of halofuginone, an oral quinazolinone derivative in patients with advanced solid tumours. *Eur J Cancer*. Aug 2006;42(12):1768-74. doi:10.1016/j.ejca.2005.12.027

8. Stecklair KP, Hamburger DR, Egorin MJ, Parise RA, Covey JM, Eiseman JL. Pharmacokinetics and tissue distribution of halofuginone (NSC 713205) in CD2F1 mice and Fischer 344 rats. *Cancer Chemother Pharmacol*. Nov 2001;48(5):375-82. doi:10.1007/s002800100367

9. Koon HB, Fingleton B, Lee JY, et al. Phase II AIDS Malignancy Consortium trial of topical halofuginone in AIDS-related Kaposi sarcoma. *J Acquir Immune Defic Syndr*. Jan 1 2011;56(1):64-8. doi:10.1097/QAI.0b013e3181fc0141
