# Supplementary material for: Halofuginone for non-hospitalized adult patients with COVID-19 a multicenter, randomized placebo-controlled phase 2 trial. The HALOS trial
Source: PLoS One. 2024 Feb 23;19(2):e0299197. doi: 10.1371/journal.pone.0299197 (PMC10889621; doi:10.1371/journal.pone.0299197)
Supplement: S4 Appendix — (PDF) [file pone.0299197.s004.pdf]

Model of follow-up telephone interviews.

Day 14 interview __/ __/ __		
After introducing himself, the interviewer will ask the following questions to the patient or legal representative:		
**If the person answering the questionnaire is responsible for the patient:**		
**Mr / Mrs. Is the research participant alive?**	☐Yes	☐No
If the answer to the question above is no, the interview ends. If so, you should proceed.		
**Today:**		
**Is the research participant hospitalized?**	☐Yes	☐ No
**If not hospitalized:**		
**Do you have any type of limitation to carry out day-to-day activities , for example working, tidying up the house, going to the market and etc ...?**	☐Yes	☐No
**Are you using supplemental oxygen at home?**	☐Yes	☐ No
**If hospitalized::**		
**Are you using oxygen for any of the devices / devices?**	☐Not using oxygen	
	☐ Oxygen by nasal catheter (regular oxygen catheter)	
	☐ High flow nasal cannula or non-invasive ventilation	
	☐Intubated (mechanical ventialtion)	
**Presented some of the symptoms today:**		
**Fever (temperature higher than 37.7°C)**	☐Yes	☐No
**Muscle pain (myalgia)**	☐Yes	☐No
**Fatigue or weakness (tiredness)**	☐Yes	☐ No
**Shortness of breath**	☐Yes	☐No
**Cough**	☐Yes	☐No
**Runny nose**	☐Yes	☐ No
**Diarrhea**	☐Yes	☐No

**Nausea**	☐Yes		☐No
**Vomiting**	☐Yes<br>How many episodes ___		☐No
**Headache**	☐Yes		☐No
**Have you had any symptoms in the last 4 days?**			
**Did you have any bleeding?**	☐Yes<br>If the response is yes, ask the patient to describe the bleeding and should classify<br>it in accordance with table A.		
	☐Minor	☐ Clinically relevant non-major	☐Major
**Did you have any other type of symptoms or adverse event?**	☐Yes<br>Which? _________		☐No

<table>
<tr><td colspan="4">Day 28 interview __/ __/ __</td></tr>
<tr><td colspan="4">After introducing himself, the interviewer will ask the following questions to the patient or legal representative:</td></tr>
<tr><td colspan="4">If the person answering the questionnaire is responsible for the patient:</td></tr>
<tr><td>Mr / Mrs. Is the research participant alive?</td><td colspan="2">☐Yes</td><td>☐No</td></tr>
<tr><td colspan="4">If the answer to the question above is no, the interview ends. If so, you should proceed.</td></tr>
<tr><td colspan="4">Since the onset of COVID symptoms:</td></tr>
<tr><td>Was there a need for hospitalization?</td><td colspan="2">☐Yes</td><td>☐ No</td></tr>
<tr><td colspan="4">Is hospitalization:</td></tr>
<tr><td>Was there a need for intubation?</td><td colspan="2">☐Yes</td><td>☐No</td></tr>
<tr><td colspan="4">Have you had any of the symptoms or adverse events in the past two weeks:</td></tr>
<tr><td>Fever (temperature higher than 37.7°C)</td><td colspan="2">☐Yes</td><td>☐No</td></tr>
<tr><td>Muscle pain (myalgia)</td><td colspan="2">☐Yes</td><td>☐No</td></tr>
<tr><td>Fatigue or weakness (tiredness)</td><td colspan="2">☐Yes</td><td>☐ No</td></tr>
<tr><td>Shortness of breath</td><td colspan="2">☐Yes</td><td>☐No</td></tr>
<tr><td>Cough</td><td colspan="2">☐Yes</td><td>☐No</td></tr>
<tr><td>Runny nose</td><td colspan="2">☐Yes</td><td>☐ No</td></tr>
<tr><td>Diarrhea</td><td colspan="2">☐Yes</td><td>☐No</td></tr>
<tr><td>Nausea</td><td colspan="2">☐Yes</td><td>☐No</td></tr>
<tr><td>Vomiting</td><td colspan="2">☐Yes
How many episodes ___</td><td>☐No</td></tr>
<tr><td>Headache</td><td colspan="2">☐Yes</td><td>☐No</td></tr>
<tr><td>Did you have any bleeding?</td><td colspan="2">☐Yes
If the response is yes, ask the patient to describe the bleeding and should classify
it in accordance with table A.</td><td></td></tr>
<tr><td></td><td>☐Minor</td><td>☐ Clinically relevant non-major</td><td>☐Major</td></tr>
<tr><td>Did you have any other type of symptoms or adverse event?</td><td colspan="2">☐Yes
Which? __________</td><td>☐No</td></tr>
</table>

Table A – Bleeding classification

Type	Definition
**Major**	Defined as clinical bleeding associated with any of the following: fatal outcome, critical site involvement (intracranial, intraspinal, intraocular, pericardial, intra-articular, intramuscular with compartmental or retroperitoneal syndrome), or clinical bleeding with a drop in hemoglobin concentration ≥2g/dL, or need for transfusion of ≥2 units of packed red blood cells or whole blood. All intracerebral (or intraparenchymal) bleeds are included in the primary analysis as hemorrhagic stroke.
**Clinically relevant non-major**	Defined as clinical bleeding that does not present major bleeding criteria, but requires medical intervention, unscheduled contact (in person or by telephone) with a doctor, temporary interruption of the study drug, pain or impairment of daily activities.
**Minor**	Defined as clinical bleeding that does not meet criteria for clinically relevant major or non-major bleeding.
