# Supplementary material for: Halofuginone for non-hospitalized adult patients with COVID-19 a multicenter, randomized placebo-controlled phase 2 trial. The HALOS trial
Source: PLoS One. 2024 Feb 23;19(2):e0299197. doi: 10.1371/journal.pone.0299197 (PMC10889621; doi:10.1371/journal.pone.0299197)
Supplement: S1 Table — (DOCX) [file pone.0299197.s005.docx]

S1 Table. Viral load measurements with available results in each group ^a^

|  | Placebo  (N = 51) | Halofuginone 0.5mg  (N = 50) | Halofuginone 1mg  (N = 52) |
| --- | --- | --- | --- |
| Viral load measurements, n (%) |  |  |  |
| Three | 36 (76.5) | 42 (84) | 46 (88.5) |
| Two | 10 (19.6) | 7 (14) | 4 (7.7) |
| One | 2 (3.9) | 0 (0%) | 1 (1.9) |
| No results available | 0 (0) | 1 (2) | 1 (1.9) |
| ^a^ Considering samples collected and analyzed | | | |
