# Supplementary material for: Halofuginone for non-hospitalized adult patients with COVID-19 a multicenter, randomized placebo-controlled phase 2 trial. The HALOS trial
Source: PLoS One. 2024 Feb 23;19(2):e0299197. doi: 10.1371/journal.pone.0299197 (PMC10889621; doi:10.1371/journal.pone.0299197)
Supplement: S2 Table — (DOCX) [file pone.0299197.s006.docx]

S2 Table. Baseline Characteristics of the Patients^a^

| Characteristic | Total  (N = 153) | Placebo  (N = 51) | Halofuginone 0.5mg  (N = 50) | Halofuginone 1mg  (N = 52) |
| --- | --- | --- | --- | --- |
| Type of vaccine received, n (%) |  |  |  |  |
| Coronavac | 57 (38) | 21 (42.9) | 21 (42) | 15 (29.4) |
| AstraZeneca | 54 (36) | 19 (38.8) | 19 (38) | 16 (31.4) |
| Pfizer | 29 (19.3) | 8 (16.3) | 6 (12) | 15 (29.4) |
| Janssen | 9 (6) | 1 (2) | 3 (6) | 5 (9.8) |
| ButanVac | 1 (0.7) | 0 (0) | 1 (2) | 0 (0) |
| Laboratory variables |  |  |  |  |
| Hematocrit (%) | 43.4 (40.9 - 45.8) | 43.8 (41.4 - 46.6) | 42.5 (40.4 - 45.5) | 42.8 (40.8 - 44.6) |
| Neutrophils count ×10^9^/L | 3.4 (2.7 - 4.8) | 3.3 (2.4 - 5.8) | 3.2 (2.7 - 4.7) | 3.6 (3.0 - 4.5) |
| Urea, mg/dL | 30 (24 - 36) | 33 (24 - 36) | 31 (26 - 35) | 27.5 (23.8 - 36) |
| Alkaline Phosphatase, U/L | 69 (60 – 84) | 71 (57 - 88) | 69 (63.25 - 84) | 67.5 (61 - 79.75) |
| Gamma-GT, U/L | 32 (23 - 51) | 30 (20 - 51) | 34 (25 - 56.75) | 29.5 (23.5 - 46.25) |
| Aspartate aminotransferase, U/L | 31.5 (26.75 – 40) | 30 (26.5 - 40.5) | 34 (29 - 42) | 31 (26 - 36.5) |
| Alanine Aminotransferase, U/L | 26.5 (20 - 46) | 26 (19 - 39) | 30 (20 - 49) | 26 (20 - 42) |
| Direct bilirubin, mg/dL | 0.3 (0.14 - 0.41) | 0.32 (0.16 - 0.4) | 0.3 (0.16 - 0.47) | 0.29 (0.1 - 0.39) |
| Indirect bilirubin, mg/dL | 0.19 (0 - 0.31) | 0.22 (0.01 - 0.3) | 0.16 (0 - 0.32) | 0.18 (0 - 0.33) |
| International Normalized Ratio (INR) | 1 (1 - 1.1) | 1 (1 - 1.1) | 1 (1 - 1.1) | 1 (1 - 1.1) |
| Activated Partial Thromboplastin Time, s | 29.1 (26.6 - 31.2) | 28.5 (26.2 - 31.2) | 29 (26.8 - 31.2) | 29.7 (27.1 - 31.6) |
| Fibrinogen mg/dL | 339 (284 – 417) | 309 (270 - 414) | 376 (324 - 440) | 324 (270 - 357) |
| Sodium mEq/L | 140 (139 – 142) | 140 (139 - 142) | 140 (139 - 141) | 140 (139 - 142) |
| Potassium mEq/L | 4.2 (4 - 4.4) | 4.25 (4 - 4.5) | 4.15 (3.9 - 4.4) | 4.15 (4 - 4.38) |
| ^a^ Continuous variables are presented as median (IQR) unless otherwise indicated. | | | | |
