# Supplementary material for: Halofuginone for non-hospitalized adult patients with COVID-19 a multicenter, randomized placebo-controlled phase 2 trial. The HALOS trial
Source: PLoS One. 2024 Feb 23;19(2):e0299197. doi: 10.1371/journal.pone.0299197 (PMC10889621; doi:10.1371/journal.pone.0299197)
Supplement: S3 Table — A. Treatment adherence in the placebo group. B. Treatment adherence in the halofuginone 0.5mg group. C. Treatment adherence in the halofuginone 1mg group. (ZIP) [file pone.0299197.s007.zip › S3B Table.docx]

S3B Table. Treatment adherence in the halofuginone 0.5mg group ^a,b,c^

| **Study Day** | **Received the medication** | **Treatment suspension (AE)** | **Treatment suspension (SAE)** | **Nonadherence for other causes^d^** | **Missing data** |
| --- | --- | --- | --- | --- | --- |
| **1** | 47/50 | 0 | 0 | 0 | 3 |
| **2** | 45/50 | 1 | 0 | 0 | 4 |
| **3** | 46/50 | 2 | 0 | 0 | 2 |
| **4** | 46/50 | 2 | 0 | 0 | 2 |
| **5** | 44/50 | 2 | 0 | 0 | 4 |
| **6** | 45/50 | 2 | 0 | 0 | 3 |
| **7** | 44/50 | 2 | 0 | 0 | 4 |
| **8** | 44/50 | 3 | 0 | 0 | 3 |
| **9** | 41/50 | 4 | 0 | 1 | 4 |
| **10** | 38/50 | 4 | 0 | 1 | 7 |
| ^a^ Evaluated using patients’ answers from the daily questionnaire.  ^b^ A total of 31 (62.0%) patients confirmed have taken all 10 halofuginone 0.5mg doses.  ^c^ A total of 42 (84.0%) patients confirmed have taken at least 8 halofuginone 0.5mg doses.  ^d^ Nonadherence for other causes: 1 patient forgot to take the medication; 1 patient did not provide the reason. | | | | | |
