# Supplementary material for: Halofuginone for non-hospitalized adult patients with COVID-19 a multicenter, randomized placebo-controlled phase 2 trial. The HALOS trial
Source: PLoS One. 2024 Feb 23;19(2):e0299197. doi: 10.1371/journal.pone.0299197 (PMC10889621; doi:10.1371/journal.pone.0299197)
Supplement: S3 Table — A. Treatment adherence in the placebo group. B. Treatment adherence in the halofuginone 0.5mg group. C. Treatment adherence in the halofuginone 1mg group. (ZIP) [file pone.0299197.s007.zip › S3C Table.docx]

S3C Table. Treatment adherence in the halofuginone 1mg group ^a,b,c^

| **Study Day** | **Received the medication** | **Treatment suspension (AE)** | **Treatment suspension (SAE)** | **Nonadherence for other causes^d^** | **Missing data** |
| --- | --- | --- | --- | --- | --- |
| 1 | 51/52 | 0 | 0 | 1 | 0 |
| 2 | 51/52 | 0 | 0 | 0 | 1 |
| 3 | 47/52 | 3 | 0 | 0 | 2 |
| 4 | 45/52 | 4 | 1 | 0 | 2 |
| 5 | 43/52 | 6 | 1 | 0 | 2 |
| 6 | 42/52 | 6 | 1 | 1 | 2 |
| 7 | 41/52 | 8 | 1 | 0 | 2 |
| 8 | 39/52 | 8 | 1 | 0 | 4 |
| 9 | 35/52 | 8 | 1 | 0 | 8 |
| 10 | 33/52 | 9 | 1 | 1 | 8 |
| ^a^ Evaluated using patients’ answers from the daily questionnaire.  ^b^ A total of 29 (55.8%) patients confirmed have taken all 10 halofuginone 1mg doses.  ^c^ A total of 39 (75.0%) patients confirmed have taken at least 8 halofuginone 1mg doses.  ^d^ Nonadherence for other causes: 3 patients forgot to take the medication. | | | | | |
