# Supplementary material for: Halofuginone for non-hospitalized adult patients with COVID-19 a multicenter, randomized placebo-controlled phase 2 trial. The HALOS trial
Source: PLoS One. 2024 Feb 23;19(2):e0299197. doi: 10.1371/journal.pone.0299197 (PMC10889621; doi:10.1371/journal.pone.0299197)
Supplement: S4 Table — (DOCX) [file pone.0299197.s008.docx]

S4 Table. Adverse Events at day 28^a^

|  | Placebo  (N = 51) | Halofuginone 0.5mg  (N = 50) | Halofuginone 1mg  (N = 52) | p-value |
| --- | --- | --- | --- | --- |
| Adverse event, n (%) |  |  |  |  |
| Odynophagia | 9 (17.6) | 5 (10) | 4 (7.7) | 0.32 |
| Abdominal pain | 2 (3.9) | 4 (8) | 9 (17.3) | 0.08 |
| Diarrhea | 7 (13.7) | 8 (16) | 11 (21.2) | 0.61 |
| Dizziness | 2 (3.9) | 3 (6) | 8 (15.4) | 0.14 |
| Anosmia | 3 (5.9) | 5 (10) | 3 (5.8) | 0.68 |
| Increased airway secretion | 5 (9.8) | 2 (4) | 3 (5.8) | 0.52 |
| Discomfort/pain in limbs | 2 (3.9) | 5 (10) | 1 (1.9) | 0.15 |
| Dyspepsia | 0 (0) | 4 (8) | 3 (5.8) | 0.11 |
| Unspecified symptom | 0 (0) | 3 (6) | 3 (5.8) | 0.22 |
| Sinusitis | 1 (2) | 3 (6) | 1 (1.9) | 0.45 |
| Urine color change | 2 (3.9) | 2 (4) | 0 (0) | 0.40 |
| Dysphonia | 1 (2) | 1 (2) | 2 (3.8) | 1 |
| Back pain | 1 (2) | 2 (4) | 1 (1.9) | 0.69 |
| Hypertension | 0 (0) | 2 (4) | 2 (3.8) | 0.47 |
| Alopecia | 0 (0) | 1 (2) | 2 (3.8) | 0.66 |
| Forgetfulness | 0 (0) | 0 (0) | 3 (5.8) | 0.11 |
| Anxiety | 2 (3.9) | 0 (0) | 1 (1.9) | 0.66 |
| Unspecified thoracic pain | 1 (2) | 2 (4) | 0 (0) | 0.21 |
| Loss of appetite | 1 (2) | 0 (0) | 2 (3.8) | 0.77 |
| Tachycardia | 2 (3.9) | 0 (0) | 1 (1.9) | 0.66 |
| Xerostomia | 0 (0) | 1 (2) | 2 (3.8) | 0.66 |
| Depressed humor | 0 (0) | 0 (0) | 2 (3.8) | 0.33 |
| Bruise | 1 (2) | 0 (0) | 1 (1.9) | 1 |
| Hyperhidrosis | 0 (0) | 1 (2) | 1 (1.9) | 0.77 |
| Hypoacusis | 0 (0) | 1 (2) | 1 (1.9) | 0.77 |
| Otalgia | 2 (3.9) | 0 (0) | 0 (0) | 0.21 |
| Unspecified menstrual changes | 1 (2) | 0 (0) | 0 (0) | 0.66 |
| Arthralgia | 0 (0) | 1 (2) | 0 (0) | 0.33 |
| Chills | 1 (2) | 0 (0) | 0 (0) | 0.66 |
| Heat sensation | 0 (0) | 0 (0) | 1 (1.9) | 1 |
| Cystitis | 0 (0) | 1 (2) | 0 (0) | 0.33 |
| Abdominal distention | 0 (0) | 0 (0) | 1 (1.9) | 1 |
| Dysuria | 0 (0) | 1 (2) | 0 (0) | 0.33 |
| Unspecified flank pain | 0 (0) | 0 (0) | 1 (1.9) | 1 |
| Unspecified pelvic pain | 0 (0) | 1 (2) | 0 (0) | 0.33 |
| Unspecified edema | 1 (2) | 0 (0) | 0 (0) | 0.66 |
| Muscular spasms | 0 (0) | 0 (0) | 1 (1.9) | 1 |
| Sneezes | 0 (0) | 0 (0) | 1 (1.9) | 1 |
| Stomatitis | 0 (0) | 0 (0) | 1 (1.9) | 1 |
| Flatulence | 1 (2) | 0 (0) | 0 (0) | 0.66 |
| Labial herpes | 0 (0) | 1 (2) | 0 (0) | 0.33 |
| Eye redness | 0 (0) | 0 (0) | 1 (1.9) | 1 |
| Upper airway inflammation | 0 (0) | 1 (2) | 0 (0) | 0.33 |
| Insomnia | 0 (0) | 0 (0) | 1 (1.9) | 1 |
| Decreased libido | 0 (0) | 0 (0) | 2 (3.8) | 0.33 |
| Lymphadenopathy | 0 (0) | 1 (2) | 0 (0) | 0.33 |
| Dry nose | 0 (0) | 0 (0) | 1 (1.9) | 1 |
| Dy eyes | 0 (0) | 0 (0) | 1 (1.9) | 1 |
| Paresthesia | 0 (0) | 1 (2) | 0 (0) | 0.33 |
| Weight loss | 0 (0) | 0 (0) | 1 (1.9) | 1 |
| Unspecified pericarditis | 1 (2) | 0 (0) | 0 (0) | 0.66 |
| Nose pain | 1 (2) | 0 (0) | 0 (0) | 0.66 |
| Thirst | 0 (0) | 1 (2) | 0 (0) | 0.33 |
| Weezing | 0 (0) | 0 (0) | 1 (1.9) | 1 |
| Restless legs syndrome | 0 (0) | 0 (0) | 1 (1.9) | 1 |
| Unspecified head symptom | 0 (0) | 1 (2) | 0 (0) | 0.33 |
| Unspecified nose symptom | 0 (0) | 1 (2) | 0 (0) | 0.33 |
| Unspecified ear symptom | 0 (0) | 1 (2) | 0 (0) | 0.33 |
| Unspecified urinary tract symptom | 0 (0) | 1 (2) | 0 (0) | 0.33 |
| Somnolence | 1 (2) | 0 (0) | 0 (0) | 0.66 |
| Tremor | 1 (2) | 0 (0) | 0 (0) | 0.66 |
| ^a^ Each patient might have more than one adverse event. | | | |  |
