# Supplementary material for: Halofuginone for non-hospitalized adult patients with COVID-19 a multicenter, randomized placebo-controlled phase 2 trial. The HALOS trial
Source: PLoS One. 2024 Feb 23;19(2):e0299197. doi: 10.1371/journal.pone.0299197 (PMC10889621; doi:10.1371/journal.pone.0299197)
Supplement: S5 Table — A. Laboratory variables on day 10. B. Laboratory variables within groups between baseline and day 10. (ZIP) [file pone.0299197.s009.zip › S5B Table.docx]

S5B Table. Laboratory variables within groups between baseline and day 10

|  | **Placebo**  **(N = 51)** | | | **Halofuginone 0.5mg**  **(N = 50)** | | | **Halofuginone 1mg**  **(N = 52)** | | |
| --- | --- | --- | --- | --- | --- | --- | --- | --- | --- |
|  | **Baseline** | **Day 10** | **p-value** | **Baseline** | **Day 10** | **p-value** | **Baseline** | **Day 10** | **p-value** |
| Hemoglobin g/dL | 14.4 (13.6 - 15.6) | 14.2 (13.3 - 15.7) | 0.42 | 13.9 (13.4 - 14.9) | 14.1 (13.6 - 14.8) | 0.96 | 14.2 (13.5 - 14.9) | 14.2 (13.2 - 14.9) | 0.45 |
| Hematocrit (%) | 43.8 (41.4 - 46.6) | 42.3 (40.2 - 46.1) | 0.23 | 42.5 (40.4 - 45.5) | 42.2 (40.7 - 44.7) | 0.63 | 42.8 (40.8 - 44.6) | 41.9 (39.8 - 44.1) | 0.22 |
| White blood cell count ×10^9^/L | 6.3 (5.2 – 8.4) | 7.6 (6.3 – 9.1) | 0.03 | 5.9 (4.9 – 7.3) | 6.7 (5.6 – 8.0) | 0.70 | 5.9 (4.9 – 7.3) | 7.1 (5.6 – 8.1) | 0.16 |
| Neutrophils count ×10^9^/L | 3.4 (2.7 - 4.8) | 4.4 (3.4 - 5.6) | 0.02 | 3.2 (2.7 - 4.7) | 3.8 (3.0 – 4.9) | 0.32 | 3.6 (3.0 - 4.5) | 4.0 (3.3 – 4.8) | 0.28 |
| Lymphocyte count ×10^9^/L | 2.1 (1.8 – 2.5) | 2.3 (1.9 – 2.7) | 0.14 | 1.8 (1.4 – 2.2) | 2.2 (1.8 – 2.7) | <0.001 | 1.8 (1.6 – 2.2) | 2.2 (1.7 – 2.5) | 0.03 |
| Platelets count ×10^9^/L | 233 (191 – 273) | 294 (268 - 344) | <0.001 | 237 (190 – 260) | 280 (248 - 340) | <0.001 | 240 (185 – 276) | 274 (235 - 340) | 0.002 |
| Urea, mg/dL | 30 (24 - 36) | 30.5 (26.2 - 36.8) | 0.79 | 31 (26 - 35) | 34 (28 - 38) | 0.06 | 27.5 (23.8 - 36) | 33 (25 - 38) | 0.31 |
| Creatinine mg/dL | 0.83 (0.72 - 0.96) | 0.82 (0.74 - 0.99) | 0.98 | 0.84 (0.75 - 0.96) | 0.91 (0.82 - 0.98) | 0.07 | 0.87 (0.75 - 0.98) | 0.89 (0.79 - 1.01) | 0.44 |
| Alkaline Phosphatase, U/L | 69 (60 – 84) | 66 (56- 75) | 0.30 | 69 (63.25 - 84) | 66 (54 – 80) | 0.08 | 67.5 (61 - 79.75) | 60 (52 - 72) | 0.01 |
| Gamma-GT, U/L | 32 (23 - 51) | 23 (18 - 55) | 0.25 | 34 (25 - 56.75) | 27 (21 - 45) | 0.13 | 29.5 (23.5 - 46.25) | 27 (19 - 36) | 0.11 |
| Aspartate aminotransferase, U/L | 31.5 (26.75 – 40) | 23 (18 - 28) | <0.001 | 34 (29 - 42) | 22 (18 - 27) | <0.001 | 31 (26 - 36.5) | 22 (20 - 26) | <0.001 |
| Alanine Aminotransferase, U/L | 26.5 (20 - 46) | 22 (16 - 38) | 0.25 | 30 (20 - 49) | 23 (15 - 35) | 0.07 | 26 (20 - 42) | 24 (16 - 37) | 0.16 |
| Direct bilirubin, mg/dL | 0.3 (0.14 - 0.41) | 0.22 (0.13 - 0.32) | 0.07 | 0.3 (0.16 - 0.47) | 0.18 (0.14 - 0.29) | 0.04 | 0.29 (0.1 - 0.39) | 0.17 (0.13 - 0.28) | 0.33 |
| Indirect bilirubin, mg/dL | 0.19 (0 - 0.31) | 0.29 (0.2 - 0.41) | 0.006 | 0.16 (0 - 0.32) | 0.27 (0.21 - 0.41) | 0.001 | 0.18 (0 - 0.33) | 0.26 (0.2 - 0.4) | 0.021 |
| C-reactive protein mg/L | 9.85 (6.43 - 20.3) | 0.46 (0.18 - 2.35) | <0.001 | 11.7 (8.25 - 26.15) | 0.5 (0.11 - 1.08) | <0.001 | 8 (5.9 - 18.3) | 0.37 (0.08 - 1.06) | <0.001 |
| International Normalized Ratio (INR) | 1 (1 - 1.1) | 1.02 (1 - 1.07) | 0.31 | 1 (1 - 1.1) | 1.02 (1 - 1.09) | 0.44 | 1 (1 - 1.1) | 1 (1 - 1.1) | 0.67 |
| Activated Partial Thromboplastin Time, s | 29.1 (26.6 - 31.2) | 30.2 (28.8 - 32.3) | 0.03 | 29 (26.8 - 31.2) | 30.2 (27.4 - 32.1) | 0.18 | 29.7 (27.1 - 31.6) | 31.2 (28.9 - 32.8) | 0.052 |
| Fibrinogen mg/dL | 339 (284 – 417) | 297 (253 - 372) | 0.09 | 376 (324 - 440) | 321 (248 - 368) | 0.001 | 324 (270 - 357) | 285 (240 - 341) | 0.04 |
| Sodium mEq/L | 140 (139 – 142) | 141 (139 - 142) | 0.43 | 140 (139 - 141) | 140 (139 - 142) | 0.37 | 140 (139 - 142) | 140 (138 - 142) | 0.48 |
| Potassium mEq/L | 4.2 (4 - 4.4) | 4.6 (4.3 - 4.7) | <0.001 | 4.15 (3.9 - 4.4) | 4.5 (4.3 - 4.7) | <0.001 | 4.15 (4 - 4.38) | 4.5 (4.2 - 4.8) | <0.001 |
