# Supplementary material for: Halofuginone for non-hospitalized adult patients with COVID-19 a multicenter, randomized placebo-controlled phase 2 trial. The HALOS trial
Source: PLoS One. 2024 Feb 23;19(2):e0299197. doi: 10.1371/journal.pone.0299197 (PMC10889621; doi:10.1371/journal.pone.0299197)
Supplement: S5 Table — A. Laboratory variables on day 10. B. Laboratory variables within groups between baseline and day 10. (ZIP) [file pone.0299197.s009.zip › S5A Table.docx]

S5A Table. Laboratory variables on day 10^a^

|  | Total  (N = 153) | Placebo  (N = 51) | Halofuginone 0.5mg  (N = 50) | Halofuginone 1mg  (N = 52) | p-value |
| --- | --- | --- | --- | --- | --- |
| Hemoglobin g/dL | 14.2 (13.4 - 14.9) | 14.2 (13.3 - 15.7) | 14.1 (13.6 - 14.8) | 14.2 (13.2 - 14.9) | 0.66 |
| Hematocrit (%) | 42.2 (40.3 - 44.8) | 42.3 (40.2 - 46.1) | 42.2 (40.7 - 44.7) | 41.9 (39.8 - 44.1) | 0.41 |
| White blood cell count ×10^9^/L | 7.1 (5.9 – 8.5) | 7.6 (6.3 – 9.1) | 6.7 (5.6 – 8.0) | 7.1 (5.6 – 8.1) | 0.03 |
| Neutrophils count ×10^9^/L | 4.1 (3.2 – 5.2) | 4.4 (3.4 - 5.6) | 3.8 (3.0 – 4.9) | 4.0 (3.3 – 4.8) | 0.07 |
| Lymphocyte count ×10^9^/L | 2.2 (1.8 – 2.7) | 2.3 (1.9 – 2.7) | 2.2 (1.8 – 2.7) | 2.2 (1.7 – 2.5) | 0.36 |
| Platelets count ×10^9^/L | 285 (246 - 343) | 294 (268 - 344) | 280 (248 - 340) | 274 (235 - 340) | 0.21 |
| Urea, mg/dL | 33 (27 - 38) | 30.5 (26.2 - 36.8) | 34 (28 - 38) | 33 (25 - 38) | 0.17 |
| Creatinine mg/dL | 0.89 (0.77 - 0.99) | 0.82 (0.74 - 0.99) | 0.91 (0.82 - 0.98) | 0.89 (0.79 - 1.01) | 0.20 |
| Alkaline Phosphatase, U/L | 64 (54 - 76) | 66 (56- 75) | 66 (54 – 80) | 60 (52 - 72) | 0.29 |
| Gamma-GT, U/L | 26 (18 - 40) | 23 (18 - 55) | 27 (21 - 45) | 27 (19 - 36) | 0.79 |
| Aspartate aminotransferase, U/L | 22 (18 - 28) | 23 (18 - 28) | 22 (18 - 27) | 22 (20 - 26) | 0.77 |
| Alanine Aminotransferase, U/L | 23 (16 - 37) | 22 (16 - 38) | 23 (15 - 35) | 24 (16 - 37) | 0.97 |
| Direct bilirubin, mg/dL | 0.18 (0.13 - 0.3) | 0.22 (0.13 - 0.32) | 0.18 (0.14 - 0.29) | 0.17 (0.13 - 0.28) | 0.65 |
| Indirect bilirubin, mg/dL | 0.28 (0.2 - 0.41) | 0.29 (0.2 - 0.41) | 0.27 (0.21 - 0.41) | 0.26 (0.2 - 0.4) | 0.82 |
| C-reactive protein mg/L | 0.46 (0.14 - 1.32) | 0.46 (0.18 - 2.35) | 0.5 (0.11 - 1.08) | 0.37 (0.08 - 1.06) | 0.39 |
| International Normalized Ratio (INR) | 1.01 (1 - 1.09) | 1.02 (1 - 1.07) | 1.02 (1 - 1.09) | 1 (1 - 1.1) | 0.67 |
| Activated Partial Thromboplastin Time, s | 30.5 (28.5 - 32.5) | 30.2 (28.8 - 32.3) | 30.2 (27.4 - 32.1) | 31.2 (28.9 - 32.8) | 0.45 |
| Fibrinogen mg/dL | 298 (248 - 365.5) | 297 (253 - 372) | 321 (248 - 368) | 285 (240 - 341) | 0.57 |
| Sodium mEq/L | 140 (139 - 142) | 141 (139 - 142) | 140 (139 - 142) | 140 (138 - 142) | 0.20 |
| Potassium mEq/L | 4.5 (4.3 - 4.73) | 4.6 (4.3 - 4.7) | 4.5 (4.3 - 4.7) | 4.5 (4.2 - 4.8) | 0.92 |
| ^a^ Continuous variables are presented as median (IQR) unless otherwise indicated. | | | | | |
