# Supplementary material for: Halofuginone for non-hospitalized adult patients with COVID-19 a multicenter, randomized placebo-controlled phase 2 trial. The HALOS trial
Source: PLoS One. 2024 Feb 23;19(2):e0299197. doi: 10.1371/journal.pone.0299197 (PMC10889621; doi:10.1371/journal.pone.0299197)
Supplement: S6 Table — (DOCX) [file pone.0299197.s010.docx]

S6 Table. Symptoms-free days up to day 10

|  |  |  |  | **Halofuginone 0.5mg vs Placebo** | | **Halofuginone 1mg vs Placebo** | |
| --- | --- | --- | --- | --- | --- | --- | --- |
| **Outcomes** | **Placebo** | **Halofuginone 0.5mg** | **Halofuginone 1mg** | **Estimate** ^a^  **(95% CI)** | **p-value** | **Estimate**  **(95% CI)** | **p-value** |
| **Symptoms-free days up to day 10, median (IQR)** |  |  |  |  |  |  |  |
| Cough | 5 (0 - 8.5) | 6 (1.2 - 8) | 7 (4 - 9) | 1.45 (0.72; 2.92) | 0.30 | 2.28 (1.15; 4.55) | 0.02 |
| Dyspnea | 10 (9 - 10) | 10 (10 - 10) | 10 (9 - 10) | 1.99 (0.84; 4.85) | 0.1 | 1.44 (0.63; 3.33) | 0.39 |
| Rhinorrhea | 9 (7 - 9) | 9 (7.2 - 9) | 9 (8 - 9) | 1.16 (0.57; 2.35) | 0.69 | 1.39 (0.69; 2.82) | 0.35 |
| Nausea | 10 (9 - 10) | 9 (7.2 - 10) | 8 (4.8 - 9) | 0.27 (0.12; 0.56) | <0.001 | 0.10 (0.04; 0.21) | <0.001 |
| Vomit | 10 (10 - 10) | 10 (9 - 10) | 9 (9 - 10) | 0.08 (0.02; 0.25) | <0.001 | 0.07 (0.01; 0.21) | <0.001 |
| Diarrhea | 10 (9 - 10) | 10 (9 - 10) | 10 (9 - 10) | 1.12 (0.50; 2.51) | 0.79 | 1.23 (0.55; 2.8) | 0.61 |
| Fever | 9 (9 - 10) | 10 (9 - 10) | 10 (9 - 10) | 1.25 (0.58; 2.71) | 0.57 | 1.19 (0.56; 2.55) | 0.64 |
| Muscle or joint pain | 9 (8 - 9) | 9 (7 - 10) | 9 (7 - 10) | 1.39 (0.70; 2.81) | 0.3 | 1.42 (0.72; 2.83) | 0.32 |
| Headache | 9 (5.5 - 9) | 7 (5 - 9) | 7 (4 - 9) | 0.78 (0.40; 1.55) | 0.48 | 0.60 (0.30; 1.19) | 0.14 |
| Fatigue | 8 (5 - 9) | 9 (5 - 10) | 8 (4.8 - 10) | 1.58 (0.80; 3.15) | 0.19 | 1.16 (0.59; 2.29) | 0.66 |
| Abbreviations: CI, confidence interval; IQR, interquartile range.  ^a^ Estimates are proportional odds ratio | | | | | | | |
