# Supplementary material for: Halofuginone for non-hospitalized adult patients with COVID-19 a multicenter, randomized placebo-controlled phase 2 trial. The HALOS trial
Source: PLoS One. 2024 Feb 23;19(2):e0299197. doi: 10.1371/journal.pone.0299197 (PMC10889621; doi:10.1371/journal.pone.0299197)
Supplement: S7 Table — (DOCX) [file pone.0299197.s011.docx]

S7 Table. Sensitivity analysis

|  |  |  |  |  | Halofuginone 0.5mg vs Placebo | | Halofuginone 1mg vs Placebo | |
| --- | --- | --- | --- | --- | --- | --- | --- | --- |
| **Primary outcome** | Placebo  (95% CI) | Halofuginone 0.5mg (95% CI) | Halofuginone 1mg (95% CI) | Effect Statistic | Estimate  (95% CI) | p-value | Estimate  (95% CI) | p-value |
| **Swab date as continuous variable ^a^** |  |  |  |  |  |  |  |  |
| SARS-CoV-2 viral load log_10_ mean change |  |  |  |  |  |  |  |  |
| Day 5 to baseline | -2.78 (-3.35; -2.2) | -2.42 (-2.99; -1.86) | -2.57 (-3.13; -2.01) | MD | 0.35 (-0.33; 1.03) | 0.41 | 0.21 (-0.47; 0.89) | 0.72 |
| Day 10 to baseline | -3.84 (-4.47; -3.2) | -3.92 (-4.54; -3.31) | -4.12 (-4.72; -3.51) | MD | -0.09 (-0.84; 0.66) | 0.95 | -0.28 (-1.02; 0.46) | 0.61 |
| **Swab date as categorical variable ^b^** |  |  |  |  |  |  |  |  |
| SARS-CoV-2 viral load log_10_ mean change |  |  |  |  |  |  |  |  |
| Day 5 to baseline | -2.62 (-3.21; -2.02) | -2.16 (-2.76; -1.55) | -2.28 (-2.87; -1.68) | MD | 0.46 (-0.26; 1.18) | 0.26 | 0.34 (-0.37; 1.05) | 0.47 |
| Day 10 to baseline | -3.67 (-4.2; -3.14) | -3.77 (-4.31; -3.24) | -4.04 (-4.56; -3.51) | MD | -0.1 (-0.74; 0.54) | 0.91 | -0.36 (-0.99; 0.27) | 0.34 |
| **Per protocol analysis ^c^** |  |  |  |  |  |  |  |  |
| SARS-CoV-2 viral load log_10_ mean decay rate at 10 days | -3.89 (-4.59; -3.2) | -4.09 (-4.81; -3.38) | -3.94 (-4.68; -3.21) | MD | -0.2 (-1.13; 0.72) | 0.848 | -0.05 (-0.99; 0.89) | 0.990 |
| Abbreviations: CI, confidence interval; MD, mean difference.  ^a^ Analysis was performed using a mixed effect model with the date of nasal swab collection as a continuous second-degree polynomial variable with random effect for the intercept and for the viral load linear decay associated to each patient.  ^b^ Analysis was performed using a linear mixed model considering the date of nasal swab collection as a categorical variable and the group variable with an interaction term between them, with random effect for the intercept and for the viral load decay associated to each patient.  ^c^ Considering only patients who confirmed received all 10 doses of the interventions or placebo (33 patients in placebo group, 31 in halofuginone 0.5mg group and 29 in the halofuginone 1mg group). | | | | | | | | |
