# Supplementary material for: Halofuginone for non-hospitalized adult patients with COVID-19 a multicenter, randomized placebo-controlled phase 2 trial. The HALOS trial
Source: PLoS One. 2024 Feb 23;19(2):e0299197. doi: 10.1371/journal.pone.0299197 (PMC10889621; doi:10.1371/journal.pone.0299197)
Supplement: S8 Table — (DOCX) [file pone.0299197.s012.docx]

S8 Table. Post hoc analysis

|  |  |  |  | **Halofuginone 0.5mg vs Placebo** | | **Halofuginone 1mg vs Placebo** | |
| --- | --- | --- | --- | --- | --- | --- | --- |
| **Outcomes** | **Placebo  (95% CI)** | **Halofuginone 0.5mg (95% CI)** | **Halofuginone 1mg (95% CI)** | **Estimate**  **(95% CI)** | **p-value** | **Estimate**  **(95% CI)** | **p-value** |
| **At Day 14, n (%)** |  |  |  |  |  |  |  |
| Any symptoms | 23/51 (45.1) | 16/50 (32) | 20/52 (38.5) | 0.57 (0.25; 1.28) | 0.18 | 0.76 (0.34; 1.67) | 0.49 |
| Respiratory Symptoms ^a^ | 13/51 (25.5) | 9/50 (18) | 6/52 (11.5) | 0.64 (0.24; 1.66) | 0.36 | 0.38 (0.12; 1.06) | 0.07 |
| Cough | 10/51 (19.6) | 8/50 (16) | 4/52 (7.7) | 0.78 (0.27; 2.17) | 0.64 | 0.34 (0.09; 1.11) | 0.09 |
| Dyspnea | 5/51 (9.8) | 0/50 (0) | 2/52 (3.8) | - | - | - | - |
| Rhinorrhea | 4/51 (7.8) | 2/50 (4) | 0/52 (0) | - | - | - | - |
| Gastrointestinal Symptoms ^b^ | 3/51 (5.9) | 1/50 (2) | 3/52 (5.8) | 0.33 (0.02; 2.65) | 0.34 | 0.98 (0.17; 5.52) | 0.98 |
| Nausea | 1/51 (2) | 0/50 (0) | 3/52 (5.8) | - | - | - | - |
| Vomit | 0/51 (0) | 0/50 (0) | 0/52 (0) | - | - | - | - |
| Diarrhea | 3/51 (5.9) | 1/50 (2) | 0/52 (0) | - | - | - | - |
| Other Symptoms ^c^ | 12/51 (23.5) | 12/50 (24) | 15/52 (28.8) | 1.03 (0.41; 2.59) | 0.96 | 1.32 (0.55; 3.23) | 0.54 |
| Fever | 2/51 (3.9) | 1/50 (2) | 0/52 (0) | - | - | - | - |
| Muscle or joint pain | 3/51 (5.9) | 2/50 (4) | 2/52 (3.8) | 0.67 (0.08; 4.19) | 0.66 | 0.64 (0.08; 4.02) | 0.63 |
| Headache | 5/51 (9.8) | 4/50 (8) | 7/52 (13.5) | 0.80 (0.19; 3.21) | 0.75 | 1.43 (0.43; 5.15) | 0.56 |
| Fatigue | 7/51 (13.7) | 7/50 (14) | 10/52 (19.2) | 1.02 (0.32; 3.23) | 0.99 | 1.50 (0.53; 4.47) | 0.45 |
| **At Day 28, n (%)** |  |  |  |  |  |  |  |
| Any symptoms | 11/51 (21.6) | 10/50 (20) | 14/52 (26.9) | 0.91 (0.34; 2.39) | 0.85 | 1.34 (0.54; 3.37) | 0.53 |
| Respiratory Symptoms ^a^ | 5/51 (9.8) | 4/50 (8) | 4/52 (7.7) | 0.80 (0.19; 3.21) | 0.75 | 0.77 (0.18; 3.07) | 0.70 |
| Cough | 4/51 (7.8) | 4/50 (8) | 4/52 (7.7) | 1.02 (0.23; 4.55) | 0.98 | 0.98 (0.22; 4.36) | 0.98 |
| Dyspnea | 2/51 (3.9) | 0/50 (0) | 1/52 (1.9) | - | - | - | - |
| Rhinorrhea | 1/51 (2) | 0/50 (0) | 1/52 (1.9) | - | - | - | - |
| Gastrointestinal Symptoms ^b^ | 1/51 (2) | 1/50 (2) | 2/52 (3.8) | 1.02 (0.04; 26.29) | 0.99 | 2.00 (0.19; 43.85) | 0.58 |
| Nausea | 0/51 (0) | 0/50 (0) | 1/52 (1.9) | - | - | - | - |
| Vomit | 0/51 (0) | 0/50 (0) | 0/52 (0) | - | - | - | - |
| Diarrhea | 1/51 (2) | 1/50 (2) | 1/52 (1.9) | 1.02 (0.04; 26.29) | 0.99 | 0.98 (0.04; 25.25) | 0.99 |
| Other Symptoms ^c^ | 7/51 (13.7) | 7/50 (14) | 11/52 (21.2) | 1.02 (0.32; 3.23) | 0.99 | 1.69 (0.61; 4.98) | 0.32 |
| Fever | 0/51 (0) | 0/50 (0) | 0/52 (0) | - | - | - | - |
| Muscle or joint pain | 3/51 (5.9) | 1/50 (2) | 2/52 (3.8) | 0.33 (0.02; 2.65) | 0.34 | 0.64 (0.08; 4.02) | 0.63 |
| Headache | 2/51 (3.9) | 2/50 (4) | 4/52 (7.7) | 1.02 (0.12; 8.79) | 0.98 | 2.04 (0.38; 15.22) | 0.42 |
| Fatigue | 6/51 (11.8) | 5/50 (10) | 9/52 (17.3) | 0.83 (0.23; 2.96) | 0.78 | 1.57 (0.52; 5.03) | 0.43 |
| Abbreviations: CI, confidence interval.  ^a^ Respiratory symptoms included: cough, dyspnea, and rhinorrhea  ^b^ Gastrointestinal symptoms included: nausea, vomit, and diarrhea  ^c^ Other symptoms included: fever, muscle or joint pain, headache, and fatigue | | | | | | | |
