# Supplementary figures and images for: Halofuginone for non-hospitalized adult patients with COVID-19 a multicenter, randomized placebo-controlled phase 2 trial. The HALOS trial
Source: PLoS One. 2024 Feb 23;19(2):e0299197. doi: 10.1371/journal.pone.0299197 (PMC10889621; doi:10.1371/journal.pone.0299197)

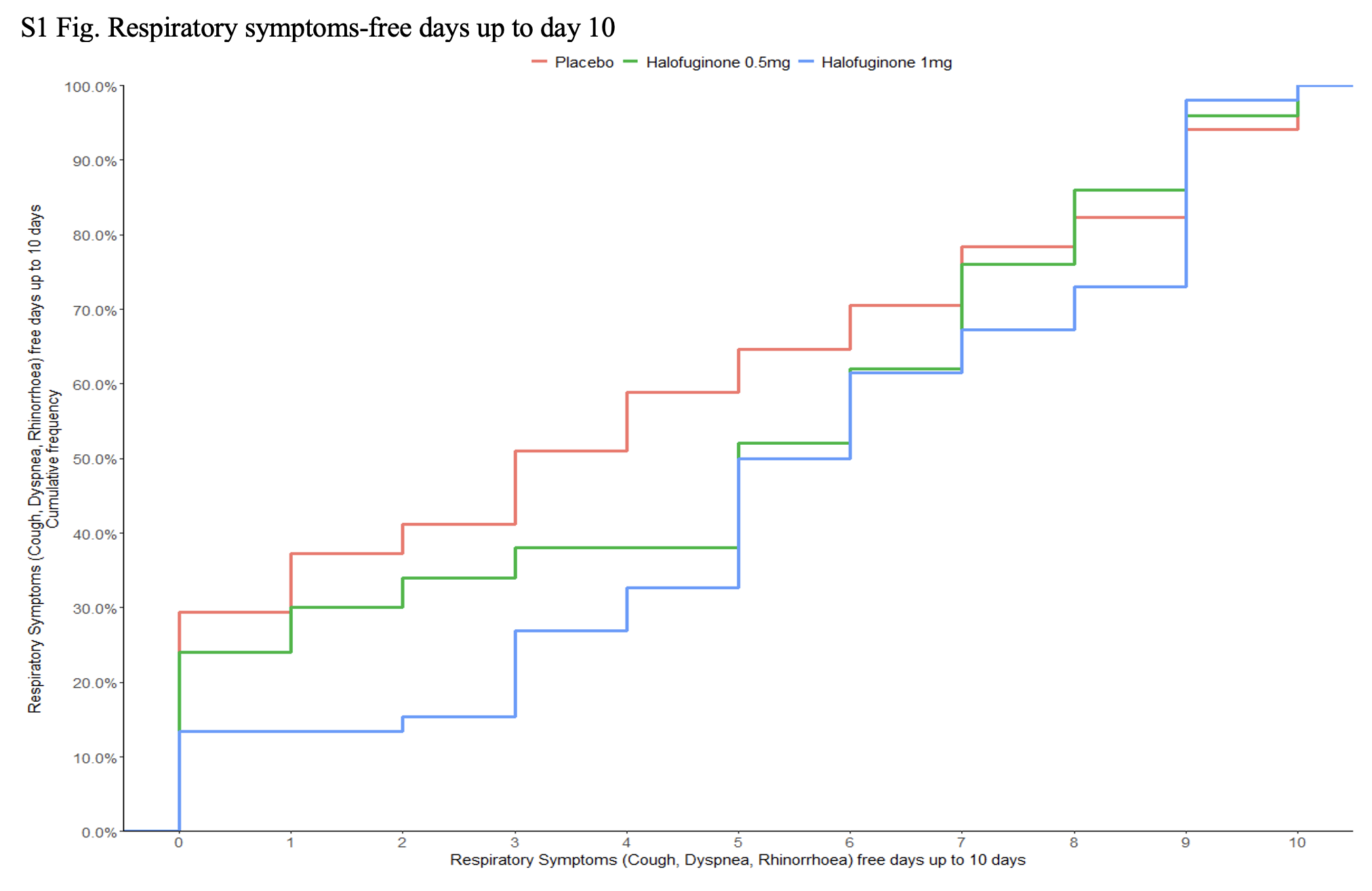

Supplement: S1 Fig — (TIF) [file pone.0299197.s013.tif]

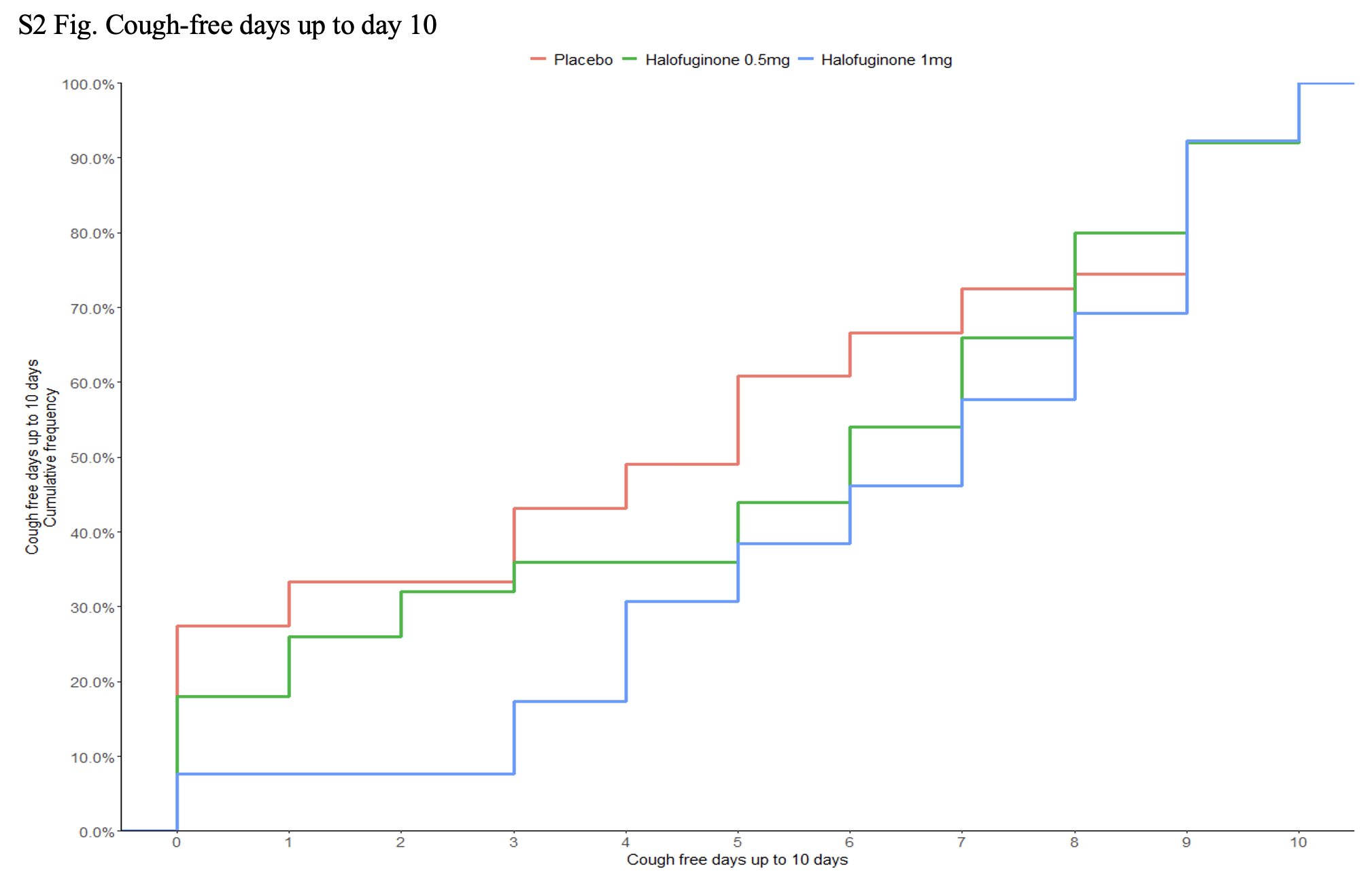

Supplement: S2 Fig — (TIF) [file pone.0299197.s014.tif]

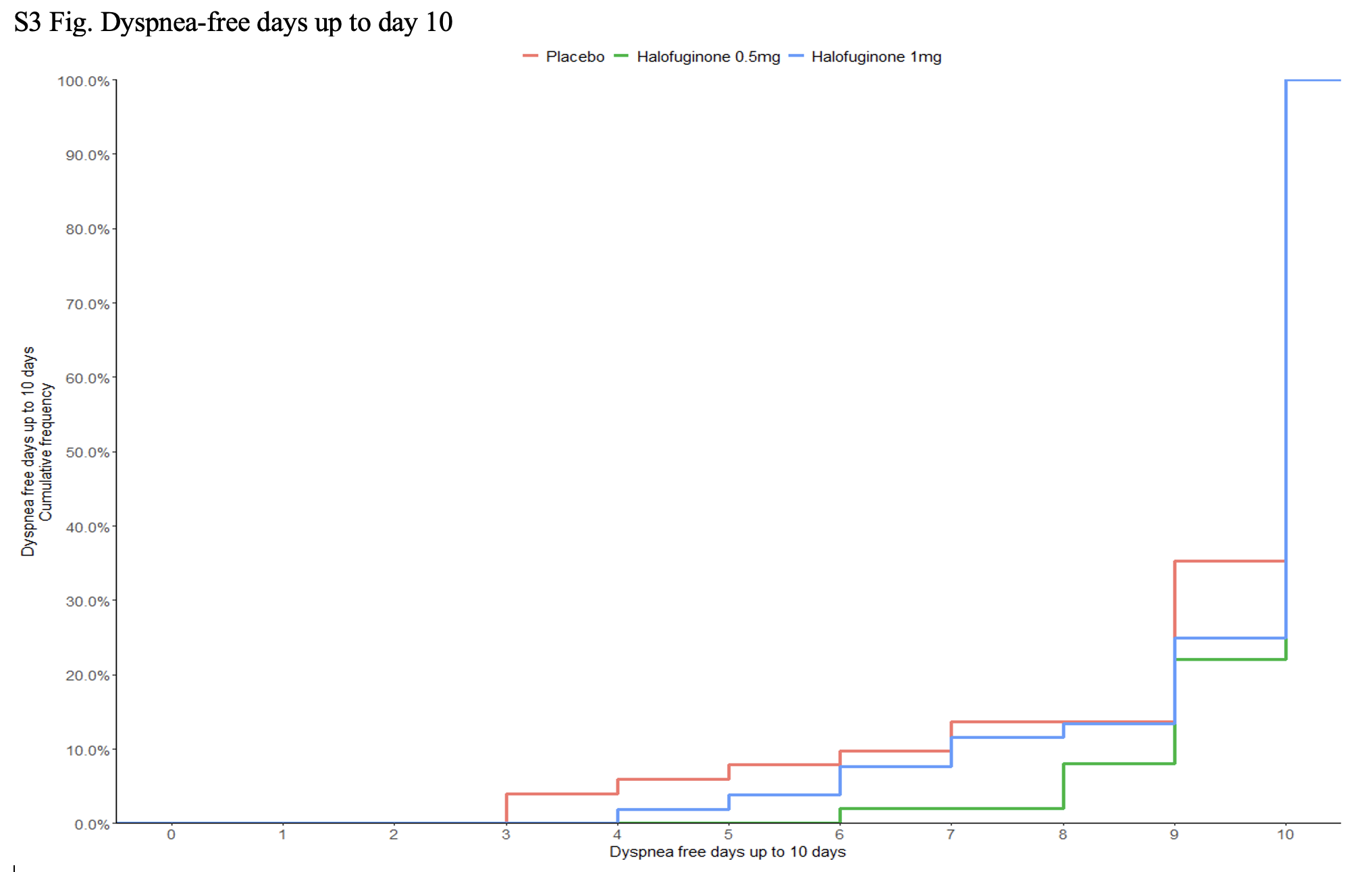

Supplement: S3 Fig — (TIF) [file pone.0299197.s015.tif]

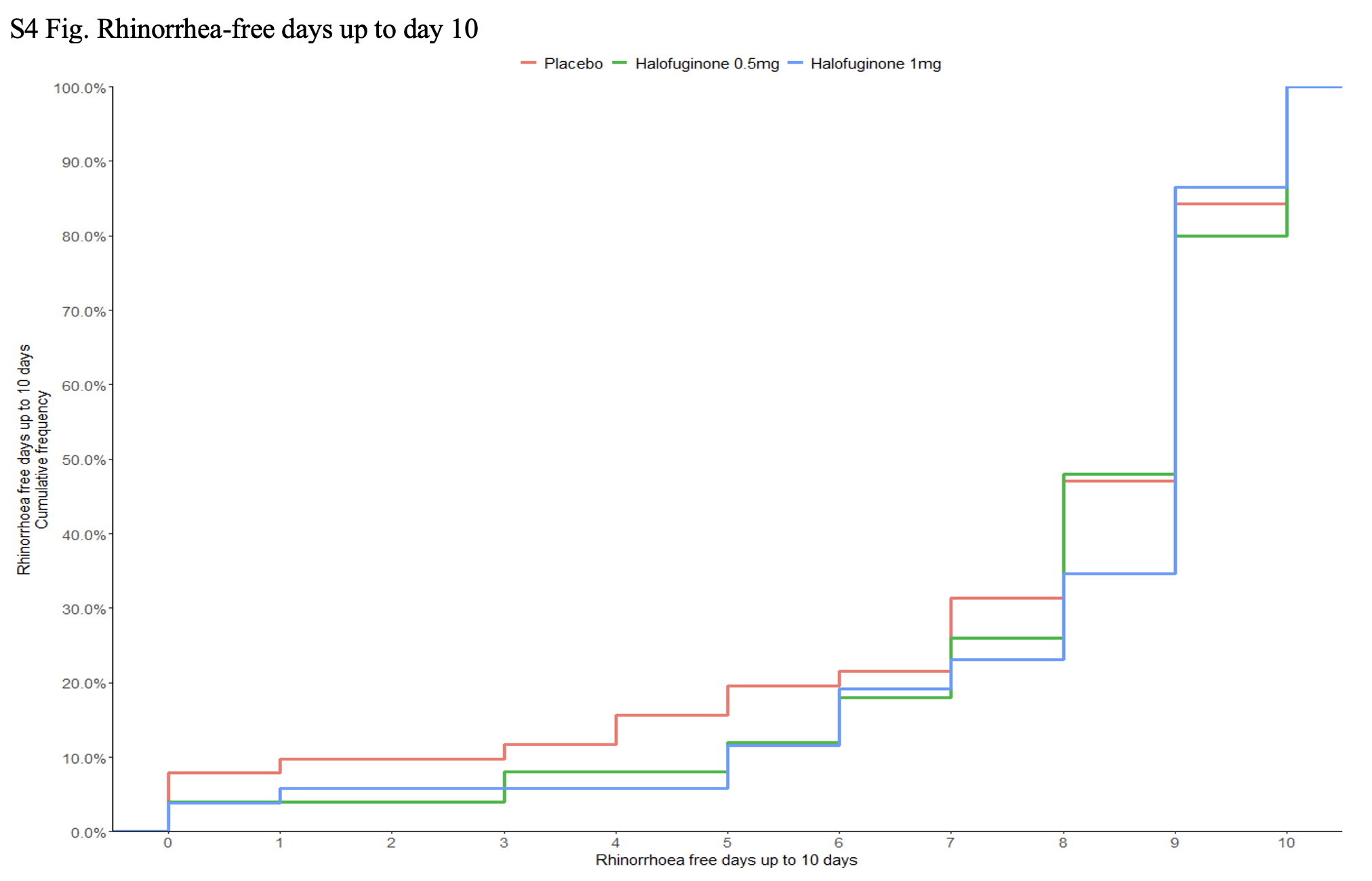

Supplement: S4 Fig — (TIF) [file pone.0299197.s016.tif]

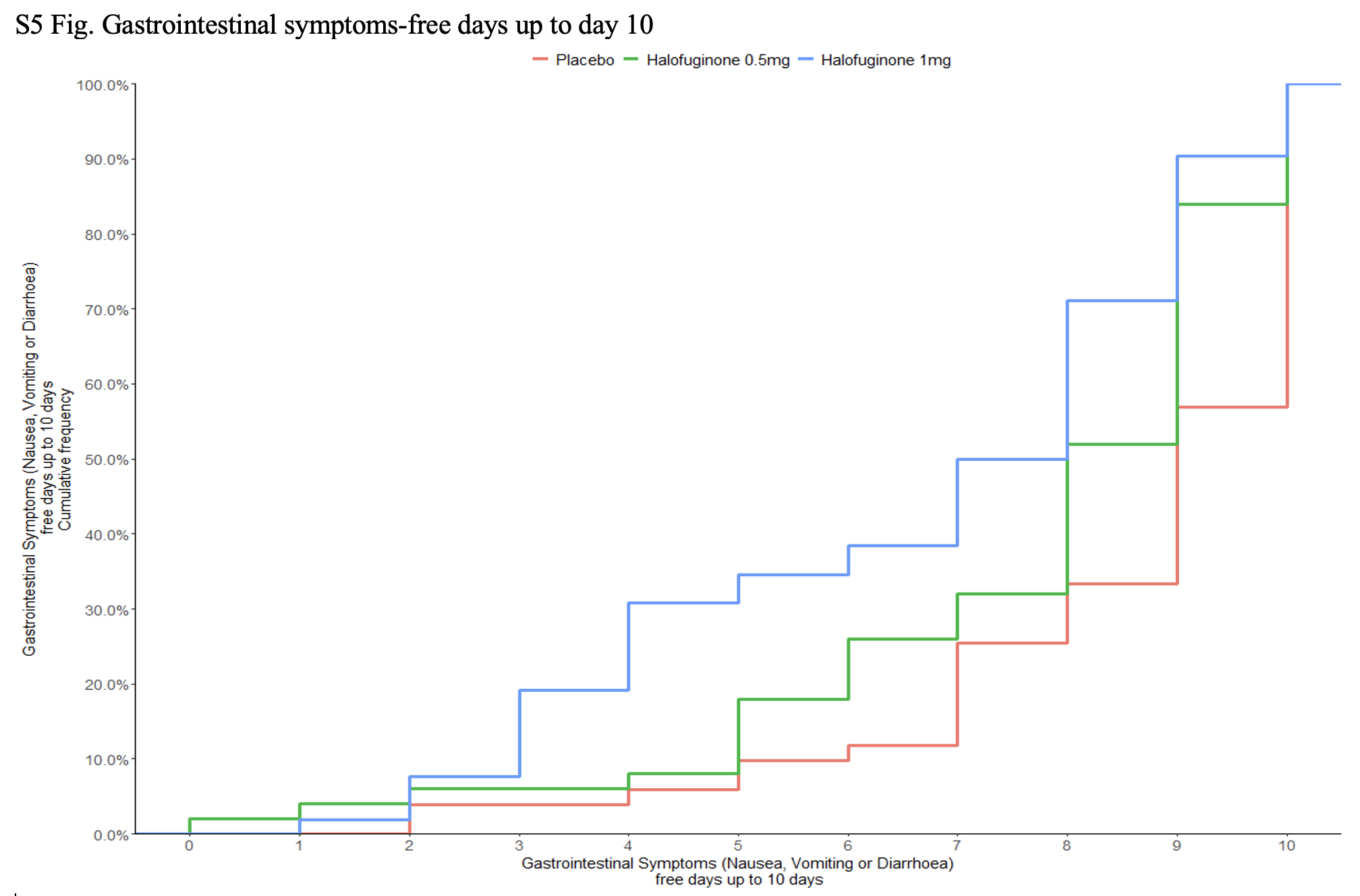

Supplement: S5 Fig — (TIF) [file pone.0299197.s017.tif]

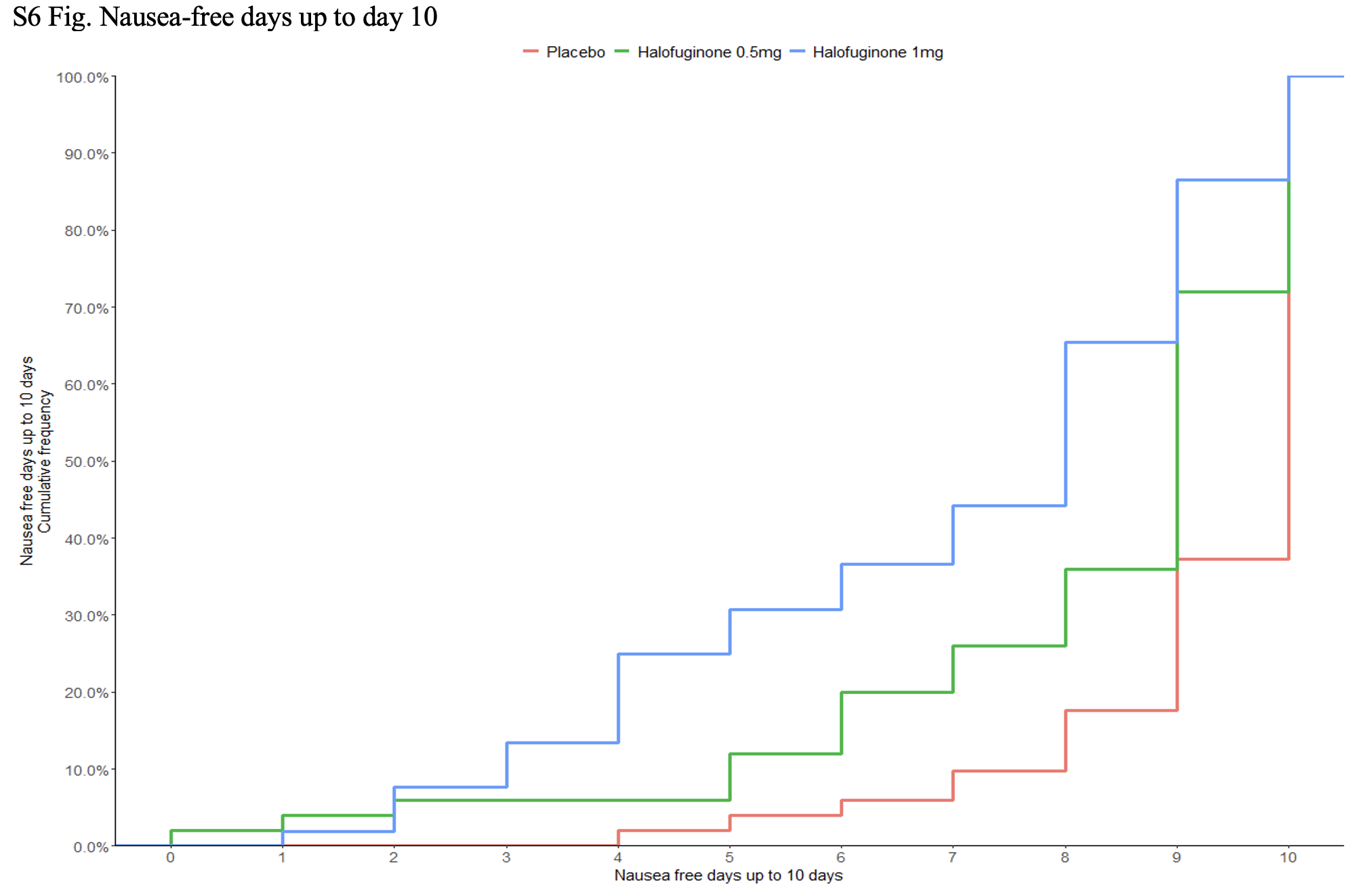

Supplement: S6 Fig — (TIF) [file pone.0299197.s018.tif]

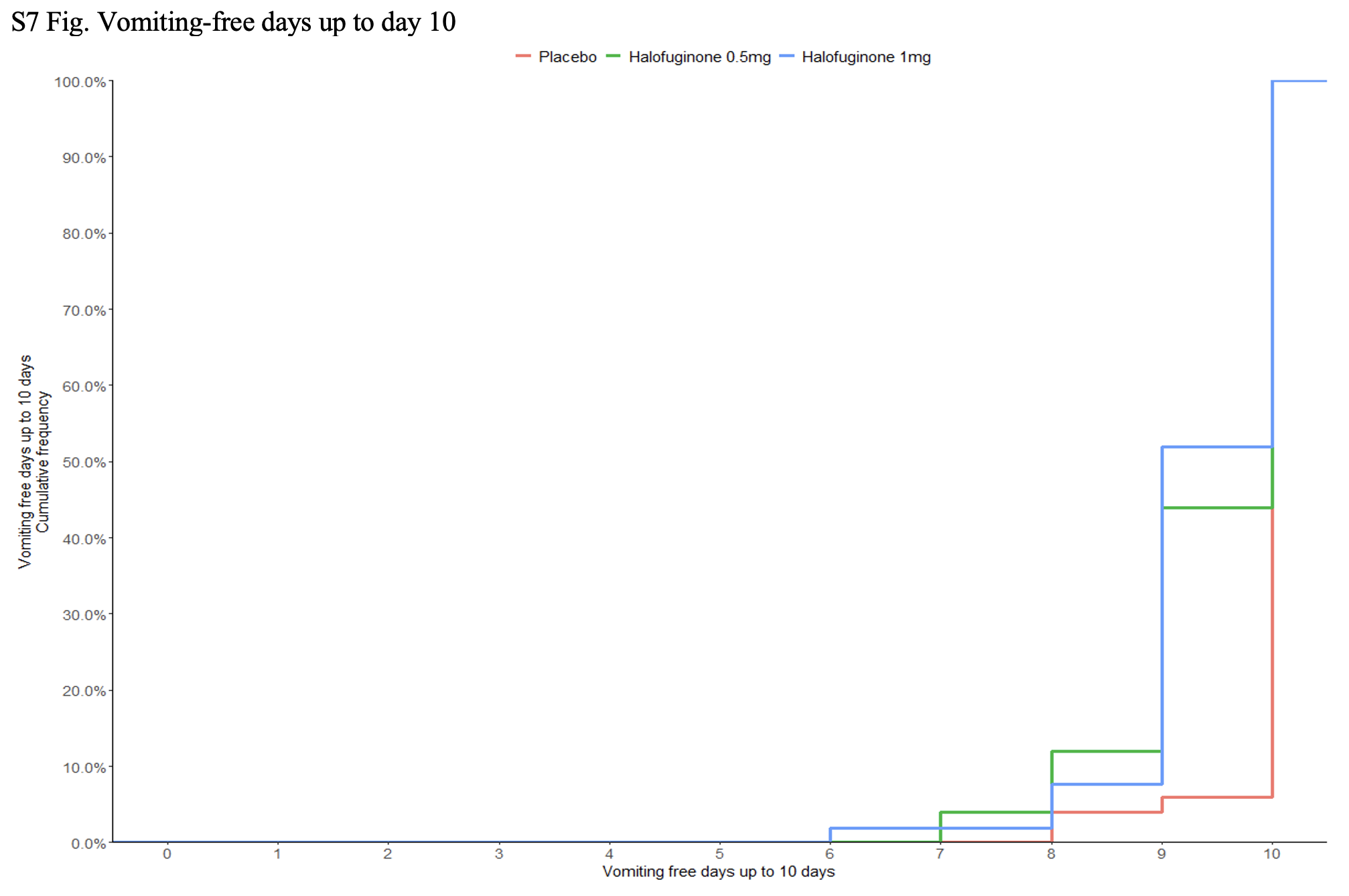

Supplement: S7 Fig — (TIF) [file pone.0299197.s019.tif]

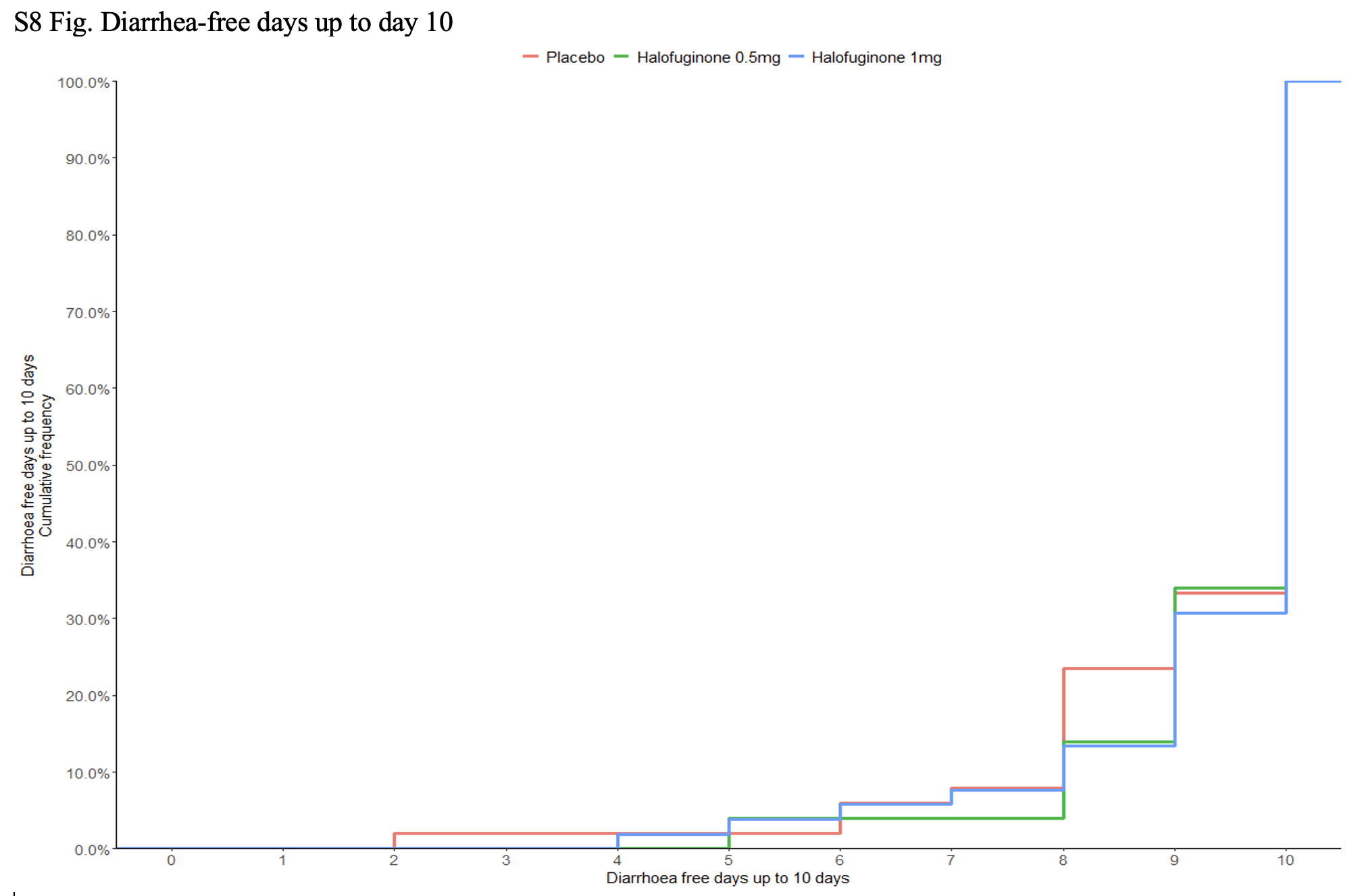

Supplement: S8 Fig — (TIF) [file pone.0299197.s020.tif]

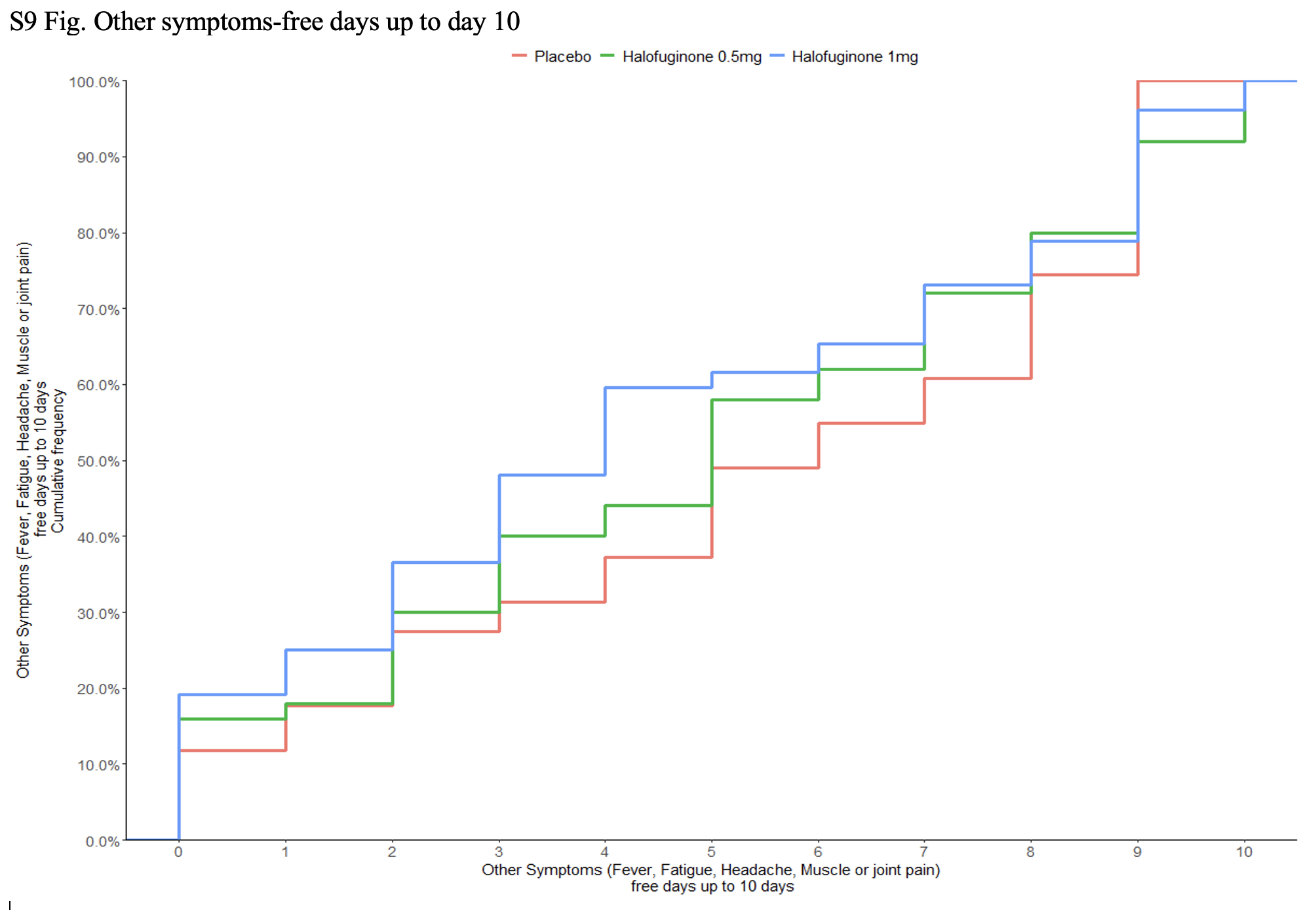

Supplement: S9 Fig — (TIF) [file pone.0299197.s021.tif]

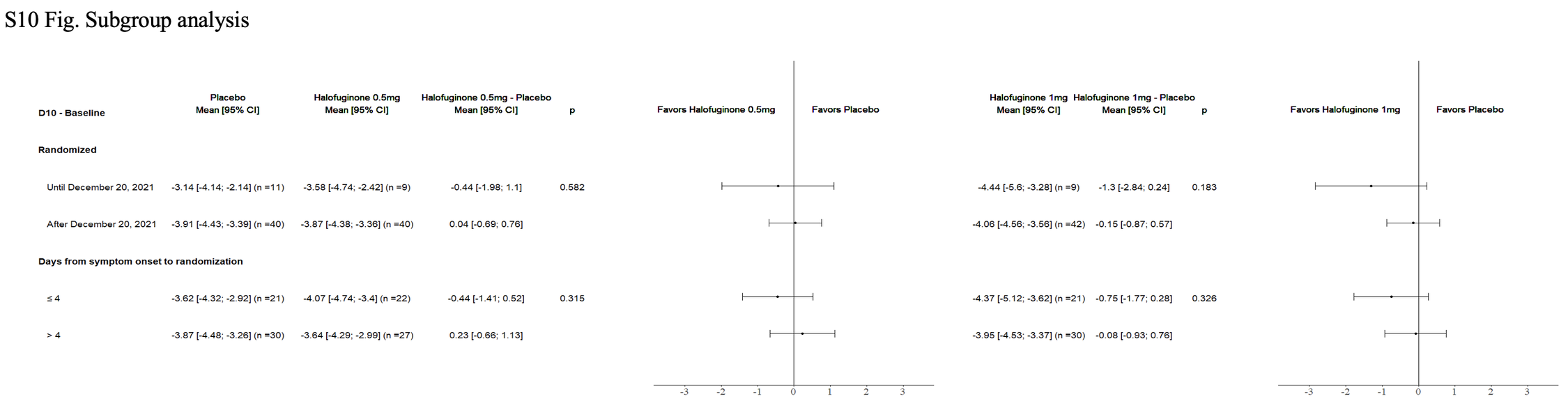

Supplement: S10 Fig — (TIF) [file pone.0299197.s022.tif]
